# Natural environment and gestational diabetes risk in Australia: a spatiotemporal ecological regression approach

**DOI:** 10.1186/s12889-026-26778-7

**Published:** 2026-03-06

**Authors:** Wubet Worku Takele, Siew Lim, Lachlan L. Dalli, Richard Beare, Kiki Adhinugraha, David Taniar, Siqin Wang, Jacqueline A. Boyle

**Affiliations:** 1 Eastern Health Clinical School, Monash University, Melbourne, Victoria Australia; 2 Stroke and Ageing Research, Department of Medicine, School of Clinical Sciences at Monash Health, Monash University, Victoria, Australia; 3 Developmental Imaging, Murdoch Children’s Research Institute, Melbourne, Australia; 4 Peninsula Clinical School, Central Clinical School, Monash University, Melbourne, VIC Australia; 5 Department of Computer Science and Information Technology, School of Engineering and Mathematical Sciences, La Trobe University, Melbourne, Victoria Australia; 6 Faculty of Information Technology, Monash University, Melbourne, Victoria Australia; 7 Spatial Sciences Institute, University of Southern California, Los Angeles, CA USA

**Keywords:** Air pollution, Environmental equity, Gestational diabetes, Greenness, Social environment

## Abstract

**Background:**

Disproportionate exposure to natural environment attributes (e.g., greenness, air quality) may be associated with disparities in gestational diabetes mellitus (GDM) risk across areas and social factors. We examined the association between the natural environment features and GDM risk, and explored variation by area-level social factors.

**Methods:**

A nationwide ecological spatiotemporal study at Statistical Area Level 2 (SA2), a medium-sized spatial resolution, was conducted using data from 2016 to 2022. SA2-level annual data on GDM cases, births, environmental exposures (air pollution [PM_2.5_ and NO_2_], greenness, and temperature), and neighbourhood social factors (e.g., socioeconomic status) were used. Residential greenness was measured using Normalised Differential Vegetation Index (NDVI). A spatiotemporal ecological regression approach supported by a Bayesian framework was applied. Effect modification analysis was conducted to explore whether the association between environmental exposures and the risk of GDM varied across different neigbourhood-level social factors.

**Results:**

We included 241,264 GDM cases among 2,035,100 women across 1,977 SA2s from 2016 to 2022. An 11% (Adjusted Risk Ratio [ARR]: 0.89 [0.83–0.95]) reduction in GDM risk was associated with an increase in residential greenness (per 0.10 increase in NDVI). This association was stronger in areas of most socioeconomic advantage (ARR: 0.70 [0.59–0.83]) and in neighbourhoods with a high concentration of non-European migrant women (ARR: 0.83 [0.76–0.89]). GDM risk was associated with high PM_2.5_ levels (> 5 µg/m^3^), with a 43% higher risk in areas of most socioeconomic disadvantage (ARR: 1.43 [1.11–1.88]) and a 23% higher risk in areas with high concentrations of non-European migrant women (ARR: 1.23 [1.05–1.45]). There was no significant association between NO_2_ and GDM risk, ARR: 1.01(95% CrI 1.00, 1.95]).

**Conclusions:**

High residential greenness may be associated with a lower risk of GDM, with potential differences by social factors. The increased GDM risk associated with high PM_2.5_ was more pronounced in areas of socioeconomic disadvantage and in areas with high concentrations of non-European migrant women. NO_2_ did not show a significant association with GDM risk. These findings suggest that geographically targeted interventions may help mitigate the risk of GDM associated with environmental exposures, particularly among vulnerable populations.

**Supplementary Information:**

The online version contains supplementary material available at 10.1186/s12889-026-26778-7.

## Introduction

Gestational diabetes mellitus (GDM) affects approximately 20 million women globally [1]. In Australia, the incidence of GDM has tripled, from 6.1% in 2011 to 19.3% in 2022 [2, 3]. GDM has reportedly been associated with long- and short-term adverse maternal health outcomes, including preterm birth, increased risk of GDM in subsequent pregnancies, and elevated risk of long-term cardiometabolic diseases, such as hypertension and type 2 diabetes [4]. GDM has intergenerational consequences, with offspring of pregnancies complicated by GDM having an elevated risk of developing various cardiometabolic conditions, including cardiovascular diseases [5]. The risk factors for GDM are multifactorial and involve complex interactions among biopsychosocial factors influenced by a wide range of environmental conditions, including natural and social environments.

There is recent evidence to show that natural environmental conditions (e.g., air quality, greenness, and temperature) of women influence the development of GDM [6]. Air pollutants, such as nitrogen dioxide (NO_2_) and fine particulate matter with a diameter of less than 2.5 micrometres (PM_2.5_), affect multiple organs, including the lungs and liver [7]. This may lead to inflammation, an imbalance in gut microbiome, oxidative stress, underlying risk factors for insulin resistance and subsequent GDM [7–9]. However, the association between air pollution and GDM remains mixed. Briefly, a meta-analysis that shows a 6% increase in GDM risk (with no significant difference by window of exposure) associated with PM_2.5_ exposure [10]. In contrast, two meta-analyses have shown no significant associations between the two common air pollutants (NO_2_ and PM_2.5_) and GDM risk [9, 10].

Air pollution interacts with environmental greenness and temperature, and their combined effects may elevate GDM risk [6]. Environmental greenness refers to the extent of land covered by grass, trees, shrubs, or other vegetation, including parks. High residential greenness may be beneficial to health, potentially by improving diverse biopsychosocial aspects of individuals, including reducing stress, exposure to environmental hazards (e.g., air pollution) and promoting healthy lifestyle behaviour [11]. A study in China (*n* = 6,807 persons) found that high greenness is associated with a 15% lower risk of GDM [12]. Non-optimal ambient temperature (mostly high) is another natural environmental-related factor associated with an elevated risk of GDM as highlighted in a recent meta-analysis of four studies *n* [13]. A mechanism possibly linking elevated ambient temperature exposure with GDM is that heat increases body fluid shift to the skin, which may result in dehydration and, consequently, insulin resistance [14].

Air pollution, low greenness, and high ambient temperature are often concentrated in areas of socioeconomic disadvantage and with a high proportion of migrant populations [15–18]. These subpopulations may be highly sensitive and have limited adaptive capacity (i.e., fewer resources or lack of awareness) to mitigate the harmful effects of adverse environments. The vulnerability of these subpopulations may also be exacerbated by limited access to maternal health care [19]. The convergence of social and natural environment inequities by geographical areas can amplify the metabolic health impacts during pregnancy, including the risk of GDM. Disproportionate exposure to natural environments across areas and subpopulations may indicate disparities in GDM risk among regions and population groups.

Currently, there is a dearth of evidence examining the impact of the natural environment on GDM risk using a geospatial approach to inform potential geographically targeted interventions and effective resource allocation. Additionally, evidence on the interaction between natural and social environmental factors (such as socioeconomic status) [20] and the risk of GDM is limited, despite the potential differential exposure, sensitivity, and adaptive capacity of subpopulations to environmental exposures [21]. Therefore, this study, for the first time in Australia, examines the association between the natural environment features (air quality, greenness, and temperature) and GDM risk in Australia at medium spatial resolution over time. We also explored the effect-modifying role of neighbourhood social environment factors on the association between natural environment attributes and GDM risk. We applied an advanced spatiotemporal ecological regression approach using multisource nationwide annual data (2016–2022) at a medium-sized geographical unit in Australia (Statistical Area Level 2 [SA2]). We found that higher residential greenness was associated with a lower risk of GDM, with a stronger association in urban areas and in areas with high concentrations of non-European migrant women. The association between high air pollution (PM_2.5_) and GDM risk was also stronger in areas with low socioeconomic status and high concentrations of non-European migrant women.

## Method and materials

### Study design and context

A nationwide ecological study was conducted at the Statistical Area Level 2 (SA2) in Australia, utilising data from multiple national sources spanning 2016 to 2022. For administrative and statistical purposes, Australia has four hierarchically structured Statistical Area Levels, and SA2 is the second most granular geographical structure, followed by Statistical Area Level 1 (SA1) [22]. There were 2,310 non-overlapping SA2s in Australia based on the 2016 report, including 18 SA2s with no established geometries, primarily designated to ‘Migratory-Offshore-Shipping’ and ‘no usual address’ codes for each state and territory. These non-spatial regions are designated for Migratory-Offshore workers and for people who transit through and temporarily reside there.

SA2 is a medium-sized Statistical Area in Australia, comprising approximately 3,000 to 25,000 people. Text S1 and Fig. S1 describe the study area. Eligible SA2s were selected by the 2016 Australian Bureau of Statistics (ABS) digital boundary shapefile (a file containing geographical coordinates by SA2), and areas with empty geometries were excluded. Remote and very remote areas were also excluded because of the inconsistent reporting of GDM cases to the diabetes registry (the data source for this study), which could introduce bias into risk estimation. Finally, 1,977 eligible SA2s were selected for each study calendar year (1,977 × 7). The detailed procedure for eligible SA2 selection is presented in Fig. S2.

### Data sources

#### Outcome data

We obtained GDM data and corresponding geographically aggregated birth records from the National Diabetes Services Scheme (NDSS) and the ABS, respectively, through a formal data access request. The data included the annual number of women aged 15 to 49 with GDM, along with their residential postcodes in Australia, based on their NDSS enrolment between January 1, 2016, and December 31, 2022. NDSS is a government initiative program established in 1987 and administered by Diabetes Australia that provides informational resources and subsidised equipment to promote the care of people with all types of diabetes. Women who are diagnosed with GDM are referred to the NDSS program for voluntary registration with the help of health professionals. We used NDSS as the preferred data source because it enables us to convert GDM data to SA2-levels based on an individual’s postcode, which is not possible with other national data sources, such as the Australian Institute of Health and Welfare (AIHW). According to the 2021–2022 report, the NDSS captured approximately 91% of GDM cases, compared with the most comprehensive national birth records from the AIHW [23, 24].

Since the denominator population (women who gave birth) data we obtained was aggregated to the SA2 level, we first mapped GDM cases aggregated at the residential postcode to SA2s using the Australian Statistical Geography Structure (ASGS) data allocation standard [25]. Such a data mapping approach is in line with our previous work [26] and another geospatial study in Australia [27]. In some instances, SA2s and postcodes correspond. Data on the annual number of women who gave birth between 2016 and 2022, aggregated to 1,977 (1,977 × 7) eligible SA2s, was sourced from ABS to utilise as a denominator population. Although it was possible to aggregate the GDM data into shorter time intervals (e.g., monthly), we did not have access to birth data at finer temporal scales than annual. Given the high spatial resolution (SA2) in our study and the rarity of GDM events per area, aggregating data below the annual level would yield excessive numbers of zero counts, which may affect the robustness of the analysis. Another challenge is that the ABS standardises (birth data source) small counts per area to maintain confidentiality, and many spatial units would have been standardised if we had used data at a finer temporal resolution, potentially introducing bias.

### Variables of the study

Outcome and exploratory variables included in this study are summarised in Table S1.

### Outcome variable

The outcome of the study was the annual count of GDM per SA2, based on women’s enrolment in the NDSS program. In the numerator dataset, the year was defined as the year of GDM diagnosis recorded in the NDSS, whereas in the denominator dataset, it was defined as the year of delivery. Our previous work describes GDM screening and diagnosis guidelines that were in place during our study period [28]. In summary, GDM diagnoses in our study period were based on the 2013 diagnosis guideline (implemented in 2014), which recommended universal screening with a 75 g oral glucose tolerance test [29].

### Exploratory variables

#### Sociodemographic variables (social environment)

SA2-level annual sociodemographic data, including the age of women (median age and grouped) and country of birth, were obtained from the ABS. Social environment in this study comprises two common social factors [30]: neighbourhood socioeconomic status and ethnic concentration. Data on the neighbourhood socioeconomic status at the SA2 level was obtained from ABS based on the 2016 Australian census-based Index of Relative Socio-economic Advantage and Disadvantage (IRSAD) [31]. The deciles were categorised into quintiles, with the first quintile (deciles 1 and 2) and the fifth quintile (deciles 9 and 10) denoting areas with the least and most socioeconomic advantage, respectively [31].

The ASGS classification was used to define urbanicity into two major categories: ‘major cities/urban’ and ‘regional’ areas [22]. Population density (number of people per square kilometre) at the SA2 level was determined solely from the 2016 ABS census report, and we repeated this over time because there are no annual reports.

Ethnic concentration of non-European migrants (henceforth referred to as ‘migrant women’) was determined at SA2 level based on country of birth. Country of birth was broadly classified into nine major groups, using the country of birth of women reported in the NDSS and ABS birth registry as a proxy indicator of ethnicity, aligning with the national standard ethnic classification [32]. Considering cultural similarities and GDM risk [24, 33], country of birth was further categorised into two groups: The first group (non-migrants) includes women born in Australia, New Zealand, the Americas (vast majority from North America), Northwest Europeans, and Southeast Europeans. The second category (non-European migrants) encompasses women born in North Africa and the Middle East, Northeast Asia, Southeast Asia, South and Central Asia, and Sub-Saharan Africa [33].

Next, the concentration of migrant women at the SA2 level was determined using a location quotient (LQ). LQ is a simple and widely used approach to calculate the representation of ethnic groups in local areas, including SA2 [34]. LQ estimates the ratio of the proportion of women in each ethnic group within each SA2 to the proportion of that same group in the reference population. The ratio of the proportion of migrant women to non-migrants was estimated to drive the LQ scores for each SA2. LQ above or equal to ‘1’ was defined as ‘high’ concentration migrant women, otherwise ‘low’ [34].

#### Natural environment measures (primary exposure variables)

Natural environment in this study encompasses residential greenness, ambient air quality measures (PM_2.5_ and NO_2_), and ambient temperature at the SA2 level [30]. The median annual measures of these environmental exposure measures per SA2 were derived. We used the median rather than the mean for NDVI because clouds, shadows, or sensor errors can produce extreme values. Median provides a less biased estimate, particularly in areas with mixed land cover, such as forests and urban areas. For similar reasons of minimising the effects of outliers and extreme values, the median was also used to estimate ambient air pollution measures and temperature at the SA2 level.

#### Residential greenness

We used satellite-driven median annual Normalised Difference Vegetation Index (NDVI) for 2016–2022 at the SA2 level, based on *Landsat 8* data in the Google Earth Engine (GEE), following a validated residential greenness retrieval method in Australia [35]. NDVI is one of the most widely used objective methods for measuring residential greenness [36]. The values range between ‘-1’ and ‘+1’, where values closer to ‘+1’ indicate areas with greater greenness. We excluded NDVI values less than ‘0’ as they do not capture greenness, and may cause confounding effects due to the potential beneficial effects of water, which has NDVI values near ‘-1’ (Text S2).

#### Air pollution measures

We sourced annual median concentrations of PM_2.5_ and NO_2_ at the SA2 level across Australia for 2016–2022. These were estimated using a national satellite-driven land-use regression model based on the predictions at the centroid of each 100 m grid cell [37, 38]. The model was developed by incorporating pertinent variables that are assumed to be factors determining air quality, including measured PM_2.5_ and NO_2_, land use data on natural and anthropogenic environmental measures (e.g., major roads, impervious surfaces, open space, tree covers), and satellite data influencing air pollution concentration [37, 38]. While the model for annual NO_2_ estimates explained 81% (root mean squared error [RMSE]: 1.4 parts per billion (ppb) spatial variability, the PM_2.5_ model explained 63%: 1 µg/m^3^ spatial variability [37, 38]. Using unique identifiers for SA2s and years, the NO_2_ and PM_2.5_ estimates were linked to the outcome and other SA2-level sociodemographic data for the corresponding years.

#### Environmental temperature

The annual median spatiotemporal temperature data at the SA2 level for 2016–2022 were derived using land surface temperature (LST) (°C). The data were retrieved from Google Earth Engine (GEE) platform using *Landsat 8* (Operational Land Imager (OLI)) [39]. LST is the most commonly used proxy indicator of ambient temperature worldwide [40, 41]. The LST values derived from *Landsat 8* data, originally available at 30 m spatial and 16-day temporal resolution, were aggregated to the SA2 level and annual scale using the median. Details about the data retrieval procedure and aggregation to the area of interest (SA2) are provided in Text S3.

### Data analysis

#### Descriptive analyses

The data on GDM cases, denominator (women who gave birth), and other independent variables were linked using SA2 unique identifiers and corresponding years. Spearman’s rank correlation tests were performed to assess correlations among continuous environmental measures. We used continuous and categorical primary exposure variables (natural environment) during modelling. To create categorical variables, greenness was classified as low (< 0.2), moderate (0.2–0.6), and high (≥ 0.6) based on the NDVI values [42]. Similarly, NO_2_ and PM_2.5_ were also classified as ‘low’ and ‘high’ based on the 2021 World Health Organisation (WHO) air quality guideline classification using 10 ppb (for NO_2_) and 5 µg/m^3^ (for PM_2.5_) as cut-offs [43]. Ambient temperature was also categorised based on tertiles.

#### Non-spatial modelling (generalised linear mixed-effect model [GLMM])

A rigorous, stepwise ecological modelling approach was followed, incorporating a different set of covariates (fixed effects) and random effects to select the best-fitting model. The models were adjusted for area-level social factors, including median age, socioeconomic status, ethnic concentration, and population density. Initially, the role of incorporating random effects (area [SA2] and time [year]) in improving regression model performance was explored using GLMM (Text S4).

Overdispersion in the count outcome data was diagnosed in the residuals of the standard Poisson model, suggesting that the Poisson assumption is violated (dispersion test = 3.75, *p* < 0.05 [44]. An additional test for excess zeros was conducted using the residual diagnostics for hierarchical regression models (DHARMa), which indicated no evidence of zero inflation (*p* > 0.05). Thus, a negative binomial regression was applied.

#### Spatiotemporal ecological regression

To better capture the spatial structure and potential spatial confounding, the non-spatial model was extended to a spatiotemporal ecological regression approach. First, spatial autocorrelation was examined using Moran’s I statistic from the regression residuals of the non-spatial model, and a p-value < 0.05 was taken as indicative of spatial autocorrelation [45]. This is due to the assumption that neighbouring areas share similar risk factors and exhibit spatial dependence (autocorrelation). Next, the neighbourhood weights matrix (W) was constructed using the Queen contiguity (binary) weights method for the spatial structure in the BYM model, an effective spatial smoothing technique that helps reduce noise or data variability by borrowing strength from surrounding areas (Text S5) [46, 47]. The ‘W’ was specified in the spatial regression models (see the R codes in the supplementary material). The model based on the Queen contiguity method was compared with the row-standardised approach, and the appropriate model was selected based on model parameters.

The expected number of GDM cases (E) per SA2 was estimated to account for variations in population size across SA2s. It was calculated using the observed counts of GDM cases and the reference population (women who gave birth) in each SA2 and year [48].

$$\:Eit=\frac{\sum\:itYit}{\sum\:itPit}Pit$$

where $\:Eit$ denotes the expected counts over time, $\:Yit$ and $\:Pit$, respectively, represent the observed counts and population at risk of GDM (women who gave birth) in each SA2 per annum.

A spatiotemporal ecological Besag-York-Mollie (BYM2) model, supported by a Bayesian hierarchical framework using the Integrated Laplace Approximation (INLA) approach, was used. The BYM2 model accounts for both the intrinsic Conditional Autoregressive (iCAR) model (structured random effect) and the Independent and Identically Distributed (IID) model (unstructured random effect) [49]. BYM2 is also helpful in improving the interpretability of findings by scaling and using penalised priors, thereby reducing the impact of spatial confounding. INLA is a recent and efficient approach to computing the Bayesian posterior distribution [50]. Studies in the same field applied this analytical approach to examine ecological factors associated with GDM [51].

Two BYM2-based spatiotemporal models that compared the parametric and nonparametric dynamic trends of GDM risk across SA2s were fitted [50, 52]. The first model (BYM2-linear) incorporated the spatial component and a parametric time trend across areas, assuming linearity in the temporal component (see Text S6 for *model specifications*). The second model (BYM2-RW1) extended the first by assuming a nonparametric dynamic trend across areas over time. This model was implemented by specifying a temporal random effect term using a random walk 1 (RW1) through a neighbouring structure. The models incorporate, but are not limited to, the spatial weight matrix (W), covariates, and spatial and temporal elements as recommended (*see Text S6*) [53].

Two separate single-pollutant models (each adjusted for sociodemographic variables) were fitted due to the strong correlation between PM_2.5_ and NO_2_ (ρ = 0.78). The association between natural environment features and the risk of GDM was assessed using the adjusted risk ratio (RR) with corresponding 95% credible intervals (CrIs). Models were compared using the deviance information criterion (DIC) and the Watanabe-Akaike Information Criterion (WAIC), and a model with the lowest values was selected. The analysis was performed using R version 4.3.

#### Effect modification analyses

Drawing on the recommendations of prior studies [11, 54] and the disparities of exposures and disproportionate GDM risks across subpopulations, an effect modification analysis was performed by neighbourhood socioeconomic status, urbanicity (major city vs. regional areas), and women’s ethnicity (neighbourhood ethnic concentration). This approach provides insight into the differential risk of GDM associated with natural environment for better targeted policy interventions. The interaction between variables was examined by including product terms for the exposures and candidate modifiers in regression models. The interpretation of the interaction effect was based on the ARR with CrIs.

#### Sensitivity analyses

A series of sensitivity analyses was undertaken to confirm the robustness of our findings. First, the association between ambient air pollution measures and GDM risk was further examined by considering the 2021 World Health Organisation air quality guideline cut-off values [43]. A similar analysis approach was performed to assess the association between greenness and GDM by categorising greenness into three levels based on NDVI values: low (< 0.2), moderate (0.2–0.6), and high (≥ 0.6) [42]. Second, to examine the potential influence of the minimal exposure to the outdoor environment and changes to GDM diagnosis guidelines during the COVID-19 lockdown, a sensitivity analysis was conducted by excluding data from 2020–2021. Third, an exposure-residual approach was applied to better address collinearity between the two pollutants, thereby identifying the independent association of each pollutant while adjusting for the other. Fourth, we applied a ‘single’ and ‘moving’ lag approach to examine the potential lag associations of environmental exposures with GDM risk. We created two lag variables for each exposure: (1) we assume the previous year’s environmental exposure has an association with the current year’s GDM risk (single lag [lag1’]) and (2) we have averaged estimates of environmental exposures recorded in the preceding year and the current year to obtain a variable for the moving lag (‘lag0’). For this part of the sensitivity analysis, we excluded the 2016 data because we lacked data for the preceding year.

Fifth, E-values were estimated to examine the impact of unobserved confounders on the observed association of natural environment features and GDM risk. E-value is defined as the minimum strength of association on the risk ratio scale that an unmeasured confounder would need to have with both the exposure (socio-environmental factors in our case) and the outcome (GDM risk), conditional on the measured covariates, to fully explain away the specific exposure and outcome association. A large E-value suggests that a considerable unmeasured confounding would be required to explain away the effect estimates. Lastly, environmental measures at a less granular spatial scale (e.g., SA3) may exhibit greater spatial heterogeneity, and the GDM risk associated with environmental variables may differ from that at the SA2 level. This phenomenon in spatial epidemiology is called a modifiable area unit problem (MAUP) [55]. To address this, a sensitivity analysis at the SA3 level was conducted to examine the potential differences in GDM risk associated with natural environments across the two spatial scales. A similar approach was applied in a previous study [18].

## Results

### Sociodemographic characteristics

Between 2016 and 2022, 2,035,100 women who gave birth across 1,977 SA2s over seven years (1,977 × 7) were included. Tables S2-3 provide descriptive summaries of sociodemographic characteristics of women who gave birth, and specifically those who developed GDM. After excluding remote and very remote areas, 241,264 GDM cases were recorded between 2016 and 2022. A notable increase in GDM cases was observed over time, from 26,708 (91.2 per 1,000 births) in 2016 to 41,938 (146.1 per 1,000 births) in 2022 (Fig. S3).

### Environmental air pollution

The median annual NO_2_ and PM_2.5_ concentrations for 2016–2022 were 5.92 (IQR: 4.51) (ppb) and 7.64 (IQR: 1.96) µg/m^3^, respectively. Disparities in air pollution across states/territories were observed, with the highest NO_2_ (6.5 ppb) and PM_2.5_ (8.1 µg/m^3^) concentrations in the state of Victoria. Conversely, Tasmania recorded the lowest NO_2_ concentration (1.0 ppb). NO_2_ and PM_2.5_ pollutants are presented by states/territories (Fig. S4a-b) and cities (Fig. S5a-b). Most women (92.1%, from 1,862/1,977 SA2s) reside in areas where PM_2.5_ pollution exceeds the current WHO air quality guidelines (< 5 µg/m^3^). Areas with high concentrations of migrant women showed greater air pollution than areas with low concentrations (7.5 vs. 2.8 ppb for NO_2_ and 8.2 vs. 6.5 µg/m^3^ for PM_2.5_). Furthermore, PM_2.5_ and NO_2_ pollution were higher in areas of the most socioeconomic advantage than in the least advantaged (Fig. S6a-b). Both pollutants vary across regions, but the inner regions of most major cities show high pollution levels (Figs. S7-8). Notably, all major cities recorded high PM_2.5_ concentrations, exceeding the 2021 WHO annual PM_2.5_ threshold of 5 µg/m³ (Fig. S8).

### Residential greenness

The overall annual median (IQR) national NDVI (a measure of greenness) was 0.39 (0.14), indicating a moderate greenness. Victoria, Western Australia, Queensland, and South Australia had lower greenness levels than the national average (Fig. S4c). The greenest cities were Sydney, Darwin, Hobart, and Canberra (Fig. S5c). Adelaide was the least green among the major cities in Australia (Fig. S5c). Disparities in greenness across neighbourhoods by socioeconomic status were not observed (Fig. S6c). Geographical variations in greenness distribution were observed, with low greenness in the inner areas of major cities (Fig. S9).

### Environmental temperature

The seven-year (2016–2022) annual median (IQR) national land surface temperature was 25.8 °C (IQR: 5.09). Darwin and Hobart were the warmest (38.7 °C) and coolest (15.9 °C) major cities, respectively (Fig. S5d). Geographical variations in ambient temperature were observed, with the highest values in the inner areas of major cities, except in Hobart, Tasmania (Fig. S10).

### Association between natural environment attributes and GDM risk

The correlation assessment suggested that NO_2_ and PM_2.5_ were strongly correlated (ρ = 0.78; Table S4). The GLMM incorporating both year and SA2 as random effects was the best-fitting model (AIC = 87,998; DIC = 87,969; Table S5). In the non-spatial analysis, an increase in greenness (per 0.10 increase in NDVI) was associated with an 18% lower GDM risk (ARR: 0.82 [95% CrIs: 0.76, 0.83]). Ambient air pollution and temperature measures were not significantly associated with GDM risk in the overall non-spatial analysis (Table S6). 

Examination of the spatial autocorrelation test suggests the need for spatial modelling (Moran’s I < 0.05), while accounting for spatial dependency. A spatiotemporal model with a parametric dynamic temporal trend (BYM-linear) was selected (WAIC = 85,058; DIC = 84,419, Table S7). The model based on the Queen contiguity (binary weights) method was selected over the row-standardised approach (Table S8). Interpretations and conclusions in this study were based on the outputs of the BYM-linear model. 

**Fig. 1:**
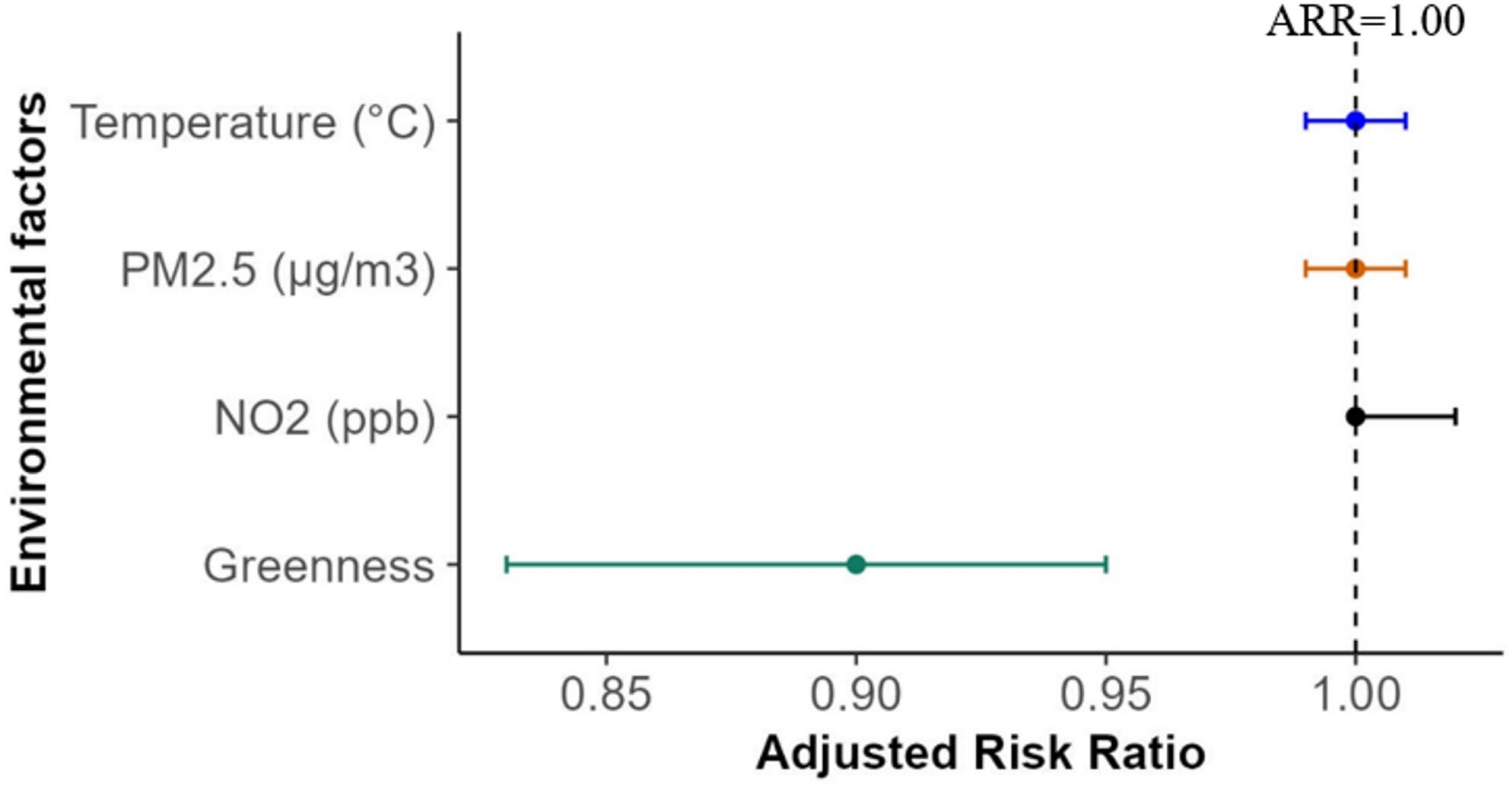
A forest plot illustrating the association between natural environmental measures and GDM risk. The model was adjusted for median age, concentration of migrant women per SA2, population density, and neighbourhood socioeconomic status. The error bars show the 95% CrIs, and the diamond shapes in the lines denote the point estimates (ARR). The estimates (ARRs with corresponding CrIs) for NO_2_ and PM_2.5_ were extracted from the single-pollutant models fitted separately (Table S7, Model 3a-b[BYM-linear) due to the high correlation between the two pollutants. The RR and CrIs for greenness and temperature were derived from a model adjusted for NO_2_ (the best-fit model, Table S7 [Model 3a]) and other factors, including socioeconomic status, ethnic concentration, remoteness, and population density

An increase in greenness (per 0.10 increase in NDVI) was associated with a 11% lower GDM risk (ARR: 0.89 [95% CrI: 0.83, 0.95]), which was attenuated compared with findings of the non-spatial analysis. No significant association between other environmental exposures (temperature, PM_2.5_, and NO_2_) and GDM risk was observed, consistent with the non-spatial model (Fig. 1).

### Effect modification

#### Association between greenness and GDM risk by neigbourhood-level social factors

**Fig. 2:**
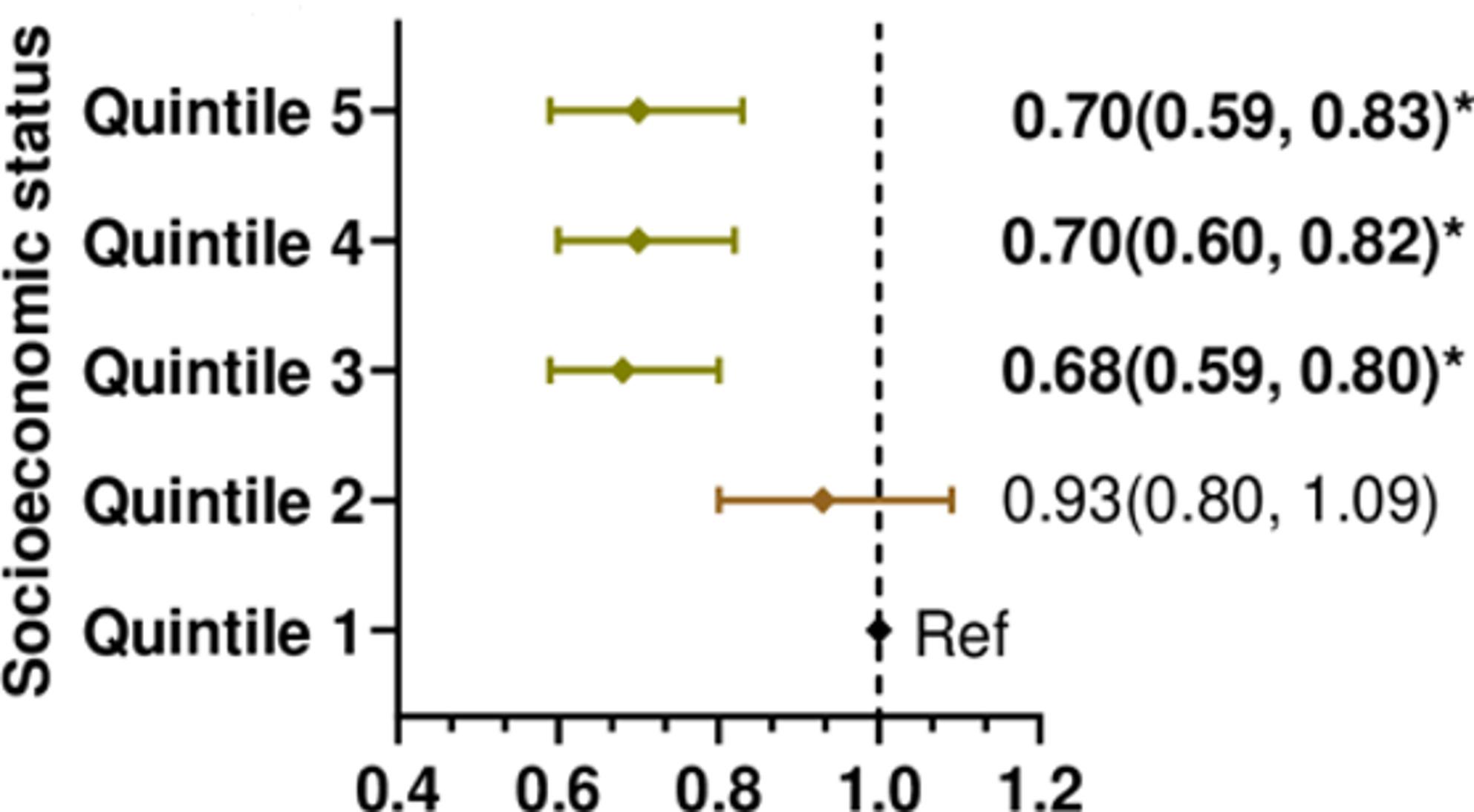
Forest plots showing the effect of modification of neighbourhood socioeconomic status on the association between greenness and GDM risk. The model incorporated an interaction term (greenness * socioeconomic status) and was adjusted for area-level median age, population density, air pollution (NO2), and temperature. Quintiles 1 and 5 represent the least and most socioeconomic advantage, respectively. ARR: Adjusted risk ratio. Ref: reference category. *denotes significant association

Effect modification analysis indicated that the greenness-GDM association differed by social factors (socioeconomic status, urbanicity, and migrant concentration) and by air pollution. Compared with areas of the most socioeconomic disadvantage, a lower GDM risk in the least disadvantaged areas was associated with areas of high (per 0.10 increase in NDVI) greenness (Fig. 2).

The association of high greenness and GDM risk was stronger in women residing in major cities (ARR: 0.87 [95% CrIs; 0.78, 0.97]) than in regional areas. Similarly, a stronger association between high greenness and GDM risk was observed in areas with a high concentration of migrant women (vs. low concentration) (ARR: 0.83 [95% CrIs: 0.76, 0.89]).

#### Association between air pollution and GDM risk by neighbourhood-level social factors

**Fig. 3:**
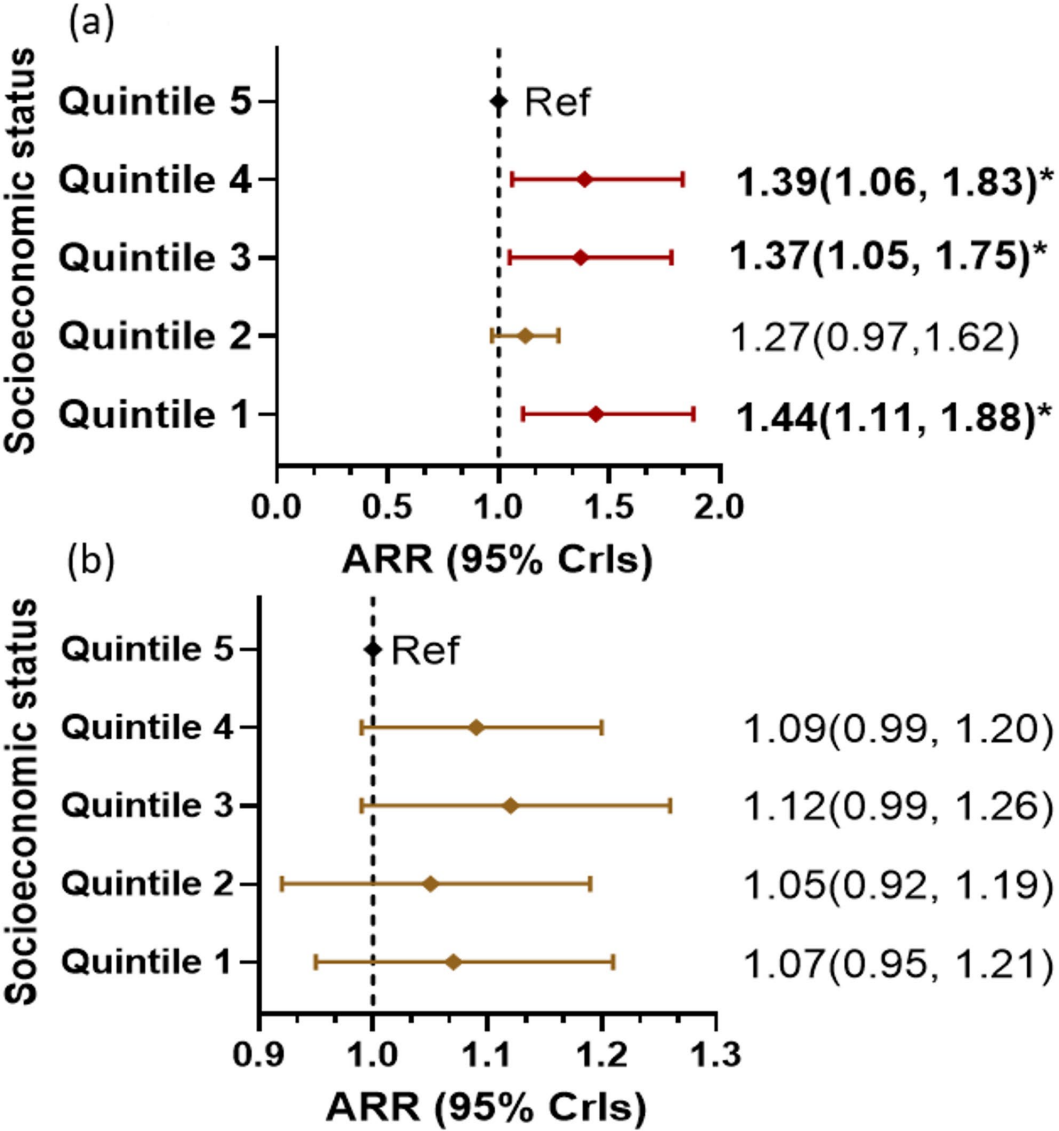
Forest plots showing the effect of modification of social environment factors on the association between air pollution and GDM risk. High PM_2.5_(>5µg/m3) vs socioeconomic status (**a**) and high NO2 (>10 ppb) vs socioeconomic status (**b**). PM_2.5_ and NO_2_ were categorised based on the 2021 WHO air quality classification. Model incorporated interaction term (potential effect modifier*exposure variable), and was adjusted for area-level median age, population density, greenness, and temperature. Low PM_2.5_ (<5µg/m3) and NO_2_ (<10 ppb) were assigned as reference categories. Ref: reference category. ARR: Adjusted risk ratio. Quintiles 1 and 5 represent the least and most socioeconomic advantage, respectively. *denotes significant association

The association between PM_2.5_ and GDM varied by socioeconomic status. An increased GDM risk was associated with high PM_2.5_ concentration (> 5 µg/m^3^) in areas of most socioeconomic disadvantage (ARR:1.44 [95% CrIs; 1.11, 1.88]) compared with the least disadvantaged areas (Fig. 3a). Similarly, GDM risk associated with high PM_2.5_ (> 5 µg/m^3^) was 23% (ARR: 1.23 [95% CrIs; 1.05, 1.45]) higher in areas with high concentrations of migrant women compared with low concentrations. Effect modification of socioeconomic status on the association between NO_2_ and GDM was not evident (Fig. 3b). Similarly, effect modification by urbanicity was not observed.

#### Sensitivity analysis

The sensitivity analysis, categorising greenness, supports the association between high greenness and a lower GDM risk, with a dose-response pattern. Briefly, a lower risk of GDM was associated with women from areas of moderate (NDVI: 0.2–0.6) and high (NDVI: ≥0.6) greenness compared with low greenness (NDVI < 0.2) (Table S9). Using the 2021 WHO air quality classification guideline, PM_2.5_ and NO_2_ concentrations were not associated with GDM risk in the overall model, consistent with findings from analyses using continuous air pollution measures (Table S9).

An additional sensitivity analysis excluding data recorded during the COVID-19 pandemic (2020–2021) shows that the association between increased greenness (per 0.10 increase in NDVI) and lower GDM risk was stronger (ARR 0.62 [95% CrIs 0.57, 0.68]; Table S10). The findings on the association between other natural environment features and GDM risk were not statistically significant, consistent with the main analysis that included data from the COVID-19 pandemic (Table S10). E-value suggests that the observed association between natural environment factors (except for PM_2.5_) and GDM risk is unlikely to be fully explained by unobserved confounders (Table S11).

Another sensitivity analysis that accounted for MAUP showed that the risk of GDM at SA3 was similar to that at SA2, indicating that greenness is associated with GDM (independent of spatial scale), whereas other natural environment measures were not significantly associated (Table S12). The residual-exposure analysis indicated no significant association between air pollutants and GDM risk, consistent with the findings from single-pollutant models (Table S13). Moreover, the estimated ‘lag’ measures of environmental exposures were not significantly associated with GDM risk (Table S14).

## Discussion

### Key findings

Our study shows that higher residential greenness may be associated with a lower risk of GDM, with potential differences by population subtypes. Women who lived in major cities, in areas of the most socioeconomic advantage, or in areas with a high concentration of non-European migrant women showed a strong association between greenness and GDM risk. Although there was no association between air pollution and GDM risk in the overall analysis, a high PM_2.5_ concentration (> 5 µg/m3) was associated with increased GDM risk among women from areas with socioeconomic disadvantage and high concentrations of non-European migrant women, which may suggest differential risk and prevention strategies.

### Greenness and GDM risk

The decreased GDM risk associated with areas with high residential greenness aligns with a previous study in the United States (US) [18] and other cohort studies in China [6, 54]. Conversely, an ecological but non-spatial study that included 362,525 women from 364 ZIP codes in the US [56] and other individual-level studies in the US and Spain have shown that greenness was not associated with the risk of GDM [57, 58]. Methodological variations arising from differences in study unit, analysis approach, population composition, and greenness measurements could explain the discrepancies in findings. For example, while the units of analysis in some studies are individuals [57, 58], area-level studies did not account for the potential spatial autocorrelations, which may have resulted in the observed variations [59]. The variable spatial scale (study units) across spatial studies may have resulted in varying GDM risks, a phenomenon known as MAUP, a common issue in spatial epidemiology [60].

The mechanism linking the lower risk of GDM in areas of high greenness remains unclear; however, it may be associated with multiple interrelated biopsychosocial and lifestyle-related factors [11]. First, high greenness may support glucose homeostasis by improving metabolic biomarkers, including insulin sensitivity, beta-cell function, and fasting glucose level [61, 62]. High greenness has also been associated with a lower risk of stress, a psychosocial factor implicated in GDM pathogenesis, by inducing inflammatory responses and insulin resistance [63, 64]. Another possible mechanism is the positive association between residential greenness and sleep quality and duration (possibly through improved mental health) [65, 66]. Residential greenness may also support enrichment of the important human gut microbiota [67], which has the potential to reduce the likelihood of developing diabetes by improving glucose metabolism [61], although further investigation is required to elucidate the underlying pathways. High residential greenness may also help mitigate the risk of GDM by promoting individuals’ physical activity and fostering social cohesion [68, 69], both of which have been shown to reduce GDM risk. Furthermore, greenness may also reduce environmental hazards, including trapping air pollutants and maintaining ambient temperature, both of which are risk factors for GDM [70]. However, the observed association between greenness and GDM risk varies across areas and subpopulations, as indicated by the effect modification analysis.

Compared with women residing in regional areas, those in major cities showed a lower risk of GDM associated with high greenness. Evidence on the differential association of greenness in improving health by urbanicity remained inconsistent [71]. For example, while a study at the individual level in China found no association, others in the pregnant and non-pregnant populations reported a lower diabetes risk associated with high residential greenness among urban residents [72]. Differences in population composition, study unit (individual-level vs. ecological), and variable ascertainment (e.g., NDVI, urbanicity) may contribute to the heterogeneous findings observed across previous studies and this work. The lower GDM risk linked to high greenness in women from urban areas could be due to highly prevalent risk factors (potentially reduced by high greenness) for diabetes in urban areas relative to rural areas. This includes greater concentrations of harmful exposures (air pollution, noise, heat) [71]. High air pollution (both NO_2_ and PM_2.5_) in major cities compared with regional areas was also observed in our study. Furthermore, the quality of greenness in urban areas may maximise its health benefits. For example, a study in Victoria, Australia, showed that some features of greenness, such as parks in urban areas, are designed to be accessible and appropriate for physical activity and recreation, with functional lighting and safety features that can promote socialisation and healthy lifestyle behaviour [73].

Similarly, women from areas of most socioeconomic advantage also showed a lower risk of GDM linked to high greenness in reducing GDM risk compared with the least advantaged areas, matching the finding of another individual-level study in the general population in China [72]. However, this evidence contradicts the findings from another individual-level study [74]. The differences could arise from differences in variable measurement (e.g., individual vs. neighbourhood level), contextual factors that require further investigation, or confounders. In our study, differences in quality, safety, and usability may contribute to the differential risk of GDM associated with greenness. For example, the sources of greenness, such as parks in areas of socioeconomic advantage, are characterised by high quality, safety, cleanliness, functionality, and accessibility [75]. Such features may more promote an individual’s recreation and physical activity behaviour, as well as frequent visits to green spaces. Moreover, the higher percentage of high-quality greenness, such as tree canopies and nearby street trees relative to roads, in most advantaged areas may provide greater benefits in reducing diabetes risk [76].

We also found disparities in the association of greenness with GDM across other social factors, demonstrating a stronger association in areas with a high concentration of migrant women (independent of socioeconomic status), similar to another study in the US [18]. Migrant women may benefit from greenness, as they are exposed to various environmental and individual-level risk factors. For example, areas with a high concentration of migrants often exhibit high air pollution, which can be potentially prevented by high greenness [77]. In addition, compared with the non-migrant population, migrants also experience other risk factors of diabetes (e.g., stress and anxiety) [78], which may be reduced by high greenness [79]. There are also hypotheses suggesting that migrants have reduced mobility and tend to spend time in green spaces, potentially gaining greater benefits. However, the mechanisms linking greenness to reduced risk of GDM, particularly among migrant women, require further investigation.

The stronger association between greenness and lower GDM risk in the sensitivity analysis that excludes data recorded during the COVID-19 pandemic remains unclear. However, it provides some insight into the greater role of greenness than we found in the overall model. The stronger association of greenness with lower GDM risk, observed after excluding data collected during the pandemic, may reflect limited opportunities for women to benefit from greenness due to restricted outdoor mobility. Furthermore, other confounders may have been present. For example, women during this period may have experienced common mental health disorders, such as stress and anxiety, that could have been reduced by greenness [79], thereby decreasing GDM risk. Additionally, health service-related confounders (e.g., disruptions to maternal healthcare services, changes in GDM screening guidelines) and behavioural changes (e.g., reduced physical activity) during COVID-19 may have attenuated the model estimates that included the COVID-19 data.

#### Air pollution and GDM risk

The association between air pollution and GDM risk in the overall model was not significant. This finding aligns with previous studies [58] but not with a spatiotemporal study in the US [51]. However, differential GDM risk associated with PM_2.5_ pollution across social factors was observed, indicating an elevated risk in areas of socioeconomic disadvantage and high concentrations of migrant women. Mechanistically, air pollution contributes to the pathogenesis of diabetes by triggering inflammation, oxidative stress, and endothelial dysfunction, thereby promoting insulin resistance.

The extent of an individual’s vulnerability to environmental hazards and associated health outcomes generally depends on three factors: exposure (the extent of the hazard and geographical location), sensitivity (presence of pre-existing aggravating risk factors), and adaptive capacity (resources and behaviours to offset the risk) [21]. Disparities in air pollution characteristics across neighbourhood socioeconomic areas may increase the likelihood of developing GDM. For example, individuals in areas of socioeconomic disadvantage are exposed to high levels of toxic air pollution, elevated emissions, and a high density of polluting industries [80]. At the individual level, the high prevalence of GDM risk factors (e.g., obesity) [81] and lack of resources to mitigate the detrimental effects of air pollution could amplify the risk of GDM among women from areas of socioeconomic disadvantage. Additionally, potential differences in awareness of the detrimental effects of air pollution and occupation type could further influence the interplay between air pollution and GDM risk in disadvantaged communities. However, the interaction between these factors and GDM risk attributable to air pollution requires further investigation.

Similarly, differences in the sensitivity and adaptive capacity of migrant women may influence their risk of developing GDM ascribed to high air pollution. For example, highly prevalent risk factors of GDM in migrant women, including unhealthy lifestyle behaviour (e.g., unhealthy diet) and low insulin sensitivity, may have exacerbated the risk of GDM attributed to air pollution [82, 83]. The lack of access to maternal health services, combined with language barriers [84] and socioeconomic constraints, could hinder healthcare utilisation and preventive measures related to air pollution exposure, which are potential confounders. Further, the high level of psychological and financial stress in migrant women may also heighten GDM risk [85]. However, due to a lack of data, we were unable to explore the interaction of these individual-level risk factors.

The pathway linking air pollution to diabetes risk remains unclear; however, it is assumed to involve insulin resistance resulting from oxidative stress, proinflammatory activation, and an imbalance between inflammation and the autonomic nervous system [7, 86]. PM_2.5_ concentration has been associated with elevated fasting oral glucose tolerance test (OGTT) in pregnant women [87]. A study in the general population has also revealed that air pollutants are associated with higher concentrations of 2-h fasting glucose, 2-h glucose, and 2-h insulin [8]. A longitudinal study involving 9,620 individuals from the general population shows that air pollution has been associated with insulin sensitivity, an important metabolic risk factor for diabetes [88].

#### Implications of the study

Despite some limitations, including confounders, the findings of this study have potential implications for environmental policies and health equity in GDM prevention. The observed association between diverse environmental features and GDM risk, with differential risk across socioeconomic and ethnic status, provides insights into efforts to tackle environmental injustice. Geographically targeted and population-specific interventions that involve stakeholders from diverse fields could be an imperative strategy. In this respect, the geographical concentration of non-European migrant women in Australia could be an opportunity to address environmental injustice effectively. In addition, most areas with socioeconomic disadvantage in Australia are places where migrants are concentrated. Therefore, this could be an additional advantage for effectively utilising resources and addressing disparities to reduce GDM risk. Importantly, given that most women (92.1%) in our study reside in areas where air pollution (PM_2.5_) exceeded the current WHO air quality guideline of 5 µg/m³, the GDM risk may continue to affect certain residents and subpopulations.

#### Strengths and limitations

To the best of our knowledge, this is the first national study to include a large sample of women in Australia, potentially making the findings generalizable. Using advanced spatiotemporal modelling, supported by a Bayesian approach, may have minimised biases arising mainly from spatial confounding and small-area-level risk estimation. This analysis approach also improves the stability of the estimates, especially in small spatial units, by sharing strength from other areas. The rigorous series of sensitivity analyses, including an assessment of GDM risk at a different spatial scale (SA3), recognising the MAUP, is the uniqueness of this study. MAUP is rarely acknowledged in spatial and spatiotemporal health studies despite variable disease risk at different spatial scales [60, 89]. The robustness of the finding was evaluated using E-value, a robust method for measuring unmeasured confounding. Additionally, this study presents new evidence on the effect-modifying role of social environment factors on the association between natural environment variables and GDM in Australia. This enabled us to understand the potential variable association between natural environment and GDM risk across social factors. This also provides new opportunities for future research to further examine the differential risks and the need for tailored policy interventions to ensure environmental health equity.

Our study has limitations that warrant caution when interpreting the findings. Data mismatch may have occurred because the numerator and denominator data were recorded across different reproductive life periods. Briefly, while GDM data are based on the year of diagnosis (during pregnancy) as reported to NDSS, the denominator data come from birth registries. For example, a woman who conceives late in the year may not deliver until the following year. However, the mismatch is likely minimal because GDM is typically diagnosed between 24 and 28 weeks of gestation. Future studies should consider using data sources that capture both GDM cases and the birth data within the same year to minimise misclassification bias and improve accuracy.

Data aggregation at the area level may have led to an ecological fallacy, masking the variability of individuals nested within areas. Similarly, due to a lack of denominator data at finer temporal resolutions (e.g., monthly), aggregating data to the annual scale may also mask differential seasonal exposures and trimester-specific exposure risks. For example, variations in GDM risk by reproductive stage (i.e., preconception and gestational age) may exist. While analysing data at a finer temporal resolution than annually offers benefits (e.g., minimising misclassification of exposure bias), it may also pose challenges for data analysis. For example, given the small number of GDM cases in smaller geographic units, i.e., SA2, further disaggregation to finer temporal resolutions could reduce the sample size for each spatial and temporal unit, potentially making the model unstable.

Another limitation is that some natural environmental metrics (e.g., NDVI) do not reflect the type, specific location (street vs. parklands), and accessibility of greenness to residents. However, it is the best objective method to quantify vegetation density globally [42]. We could not examine the differences by vegetation type (e.g. canopy, grass coverage), quality, and individual green space utilisation behaviour at the SA2 level. In addition, our model was not adjusted for confounders, including individual-level behaviours (e.g., occupation, time spent indoors/outdoors, type of energy source used for cooking). Since outdoor sources account for only 44% and 74% of individual exposure to PM_2.5_ and NO_2_, respectively [90].

Additional indoor air quality analysis might have provided further insight. However, a study has shown a strong agreement between PM_2.5_ measured using personal environmental monitors and ambient concentrations [90]. We also lacked data on the duration of residence within the same SA2, which could have introduced misclassification bias. However, the impact of migration may be minimal in this small segment of the population, and migration was negligible during the COVID-19 pandemic.

We also acknowledge that using country of birth as a proxy indicator of ethnicity has limitations. For example, this approach does not account for descendants born in Australia of parents born in these countries, and the categorisation is broad; however, it is the most common method used in Australia and globally [32, 91]. Another limitation is that we used cross-sectional reports for some potential confounders (e.g., population density) as time-variant because of the lack of annually reported data. Moreover, the allocation of population across strata in the effect modification analysis may compromise statistical power and yield biased estimates due to small sample sizes. Future studies may benefit from applying a machine learning approach to handle the complex interactions while accounting for the effect of small sample sizes.

## Conclusions

High residential greenness was associated with a lower risk of GDM. The association between increased greenness and lower GDM risk may vary across subpopulations, being stronger in areas of socioeconomic advantage, in areas with high concentrations of non-European migrant women, or in major cities. Similarly, the association between air pollution (PM_2.5_) and GDM risk may vary by social factors, with a greater GDM risk in areas of socioeconomic disadvantage and a high concentration of non-European migrant women. The findings may indicate the relevance of geographically targeted, equitable interventions to mitigate the risk of GDM associated with residential greenness and air pollution, with particular attention to vulnerable populations. The findings from this study also suggest the need for future studies that account for additional individual- and environmental-level factors.

## Supplementary Information

Supplementary Material 1.

## Data Availability

Due to ethical and legal restrictions, data from this study cannot be shared. However, aggregated data outputs and coding that support the findings of this study are available from the corresponding author upon reasonable request, subject to approval from the relevant data custodians.
