# Supplementary material for: Natural environment and gestational diabetes risk in Australia: a spatiotemporal ecological regression approach
Source: BMC Public Health. 2026 Mar 6;26:1206. doi: 10.1186/s12889-026-26778-7 (PMC13078036; doi:10.1186/s12889-026-26778-7)
Supplement: Supplementary file 1 — Supplementary Material 1. [file 12889_2026_26778_MOESM1_ESM.docx]

**Supplementary material for:**

**Natural environment and gestational diabetes risk in Australia: a spatiotemporal ecological regression approach**

Wubet Worku Takele^1^, Siew Lim^1^, Lachlan L. Dalli^2^, Richard Beare^3,4^, Kiki Adhinugraha^5^, David Taniar^6^, Siqin Wang^7^, Jacqueline A. Boyle^1*^

^1^Eastern Health Clinical School, Monash University, Melbourne, Victoria, Australia

^2^Stroke and Ageing Research, Department of Medicine, School of Clinical Sciences at Monash Health, Monash University, Victoria, Australia

^3^Developmental Imaging, Murdoch Children’s Research Institute, Melbourne, Australia

^4^Peninsula Clinical School, Central Clinical School, Monash University, Melbourne, VIC, Australia

^5^Department of Computer Science and Information Technology, School of Engineering and Mathematical Sciences, La Trobe University, Melbourne, Victoria, Australia

^6^Faculty of Information Technology, Monash University, Melbourne, Victoria, Australia.

^7^Spatial Sciences Institute, University of Southern California, Los Angeles, CA, United States

*Corresponding author

Email: [jacqueline.boyle@monash.edu](mailto:jacqueline.boyle@monash.edu)

**Contents**

[**List of Tables** 3](#_Toc216346547)

[**List of Figures** 4](#_Toc216346548)

[Text S1: Description of the study area 5](#_Toc216346549)

[Text S2: Measurement of environmental greenness 10](#_Toc216346550)

[Text S3: Measurement of environmental temperature 10](#_Toc216346551)

[Text S4: Generalised linear mixed-effect model (GLMM) 11](#_Toc216346552)

[Text S5: Spatial autocorrelation test and neighbourhood matrix construction 11](#_Toc216346553)

[Text S6: Spatiotemporal model specifications 12](#_Toc216346554)

[Text S7: Disparities in natural environment attributes across major cities 18](#_Toc216346555)

[Text S8: Codes for non-spatial (GLMM) and spatiotemporal models 28](#_Toc216346556)

[Text S9: R codes for the non-spatial and spatiotemporal models 28](#_Toc216346557)

[References 30](#_Toc216346558)

**List of Tables**

[Table S1: Measures of outcome and exploratory variables 9](#_Toc216346559)

[Table S2: Summary of sociodemographic characteristics of women who gave birth in Australia over time, 2016-222 (N=1,977 SA2s). 13](#_Toc216346560)

[Table S3: Summary of sociodemographic characteristics of women with GDM in Australia over time, 2016-2022 (N=1,977 SA2s). 15](#_Toc216346561)

[Table S4: Summary of Spearman correlation between natural environment measures. 25](#_Toc216346562)

[Table S5: Non-spatial models (generalised linear mixed-effect models [GLMMs]). 25](#_Toc216346563)

[Table S6: Summary of the non-spatial modelling on the association of natural environment and GDM risk (estimates from Model 2, Table S5). 25](#_Toc216346564)

[Table S7: Spatiotemporal ecological model comparison, 2016-2022. 25](#_Toc216346565)

[Table S8: A table comparing the BYM-based Queen contiguity (binary) and row-standardised weights. 26](#_Toc216346566)

[Table S9: Sensitivity analysis by categorical exposures (air pollution measures and greenness). 26](#_Toc216346567)

[Table S10: Sensitivity analysis showing the association between natural environment and GDM, excluding data during the COVID-19 pandemic (2020-2021). 26](#_Toc216346568)

[Table S11: Summary of E-values for the association of different natural environment measures and GDM risk. 27](#_Toc216346569)

[Table S12: Sensitivity analysis showing the association between natural environment and GDM risk at the SA3 level (checking modifiable areas unit problem [MAUP]) 27](#_Toc216346570)

[Table S13: Sensitivity analysis using the residual-exposure approach. 27](#_Toc216346571)

[Table S14: Sensitivity analysis showing the association between the lag association of environmental exposures and GDM risk. 27](#_Toc216346572)

[Table S15: Variable labels and expressions. 28](#_Toc216346573)

**List of Figures**

[Fig. S1: Study area map 6](#_Toc216346574)

[Fig. S2: A flow diagram illustrating the SA2 selection procedure 7](#_Toc216346575)

[Fig. S3: Trends in the crude incidence of GDM between 2016 and 2022. 8](#_Toc216346576)

[Fig. S4: Disparities in the natural environment characteristics by states/territories. 17](#_Toc216346577)

[Fig. S5: Violin plots illustrating the disparities in natural environment characteristics by major cities. 19](#_Toc216346578)

[Fig. S6: Disparities in natural environment characteristics by socioeconomic status. 20](#_Toc216346579)

[Fig. S7: Geographical distribution of air pollution (NO_2_) at the SA2 level in Australia over 2016-2022. 21](#_Toc216346580)

[Fig. S8: Geographical distribution of air pollution (PM_2.5_) at the SA2 level in Australia over 2016-2022. 22](#_Toc216346581)

[Fig. S9: Geographical distribution of residential greenness (based on NDVI) at the SA2 level in Australia over 2016-2022. 23](#_Toc216346582)

[Fig. S10: Geographical distribution of ambient temperature at the SA2 level in Australia over 2016-2022. 24](#_Toc216346583)

**Text S1: Description of the study area**

Australia has six states (New South Wales, Victoria, Queensland, Western Australia, South Australia, and Tasmania) and two Territories (the Australian Capital Territory and the Northern Territory). The major cities represent the capital of each state/territory: Sydney (New South Wales), Melbourne (Victoria), Brisbane (Queensland), Perth (Western Australia), Canberra (Australian Capital Territory), Adelaide (South Australia), Hobart (Tasmania), and Darwin (Northern Territory). The figure below illustrates the study area (Fig. S1).


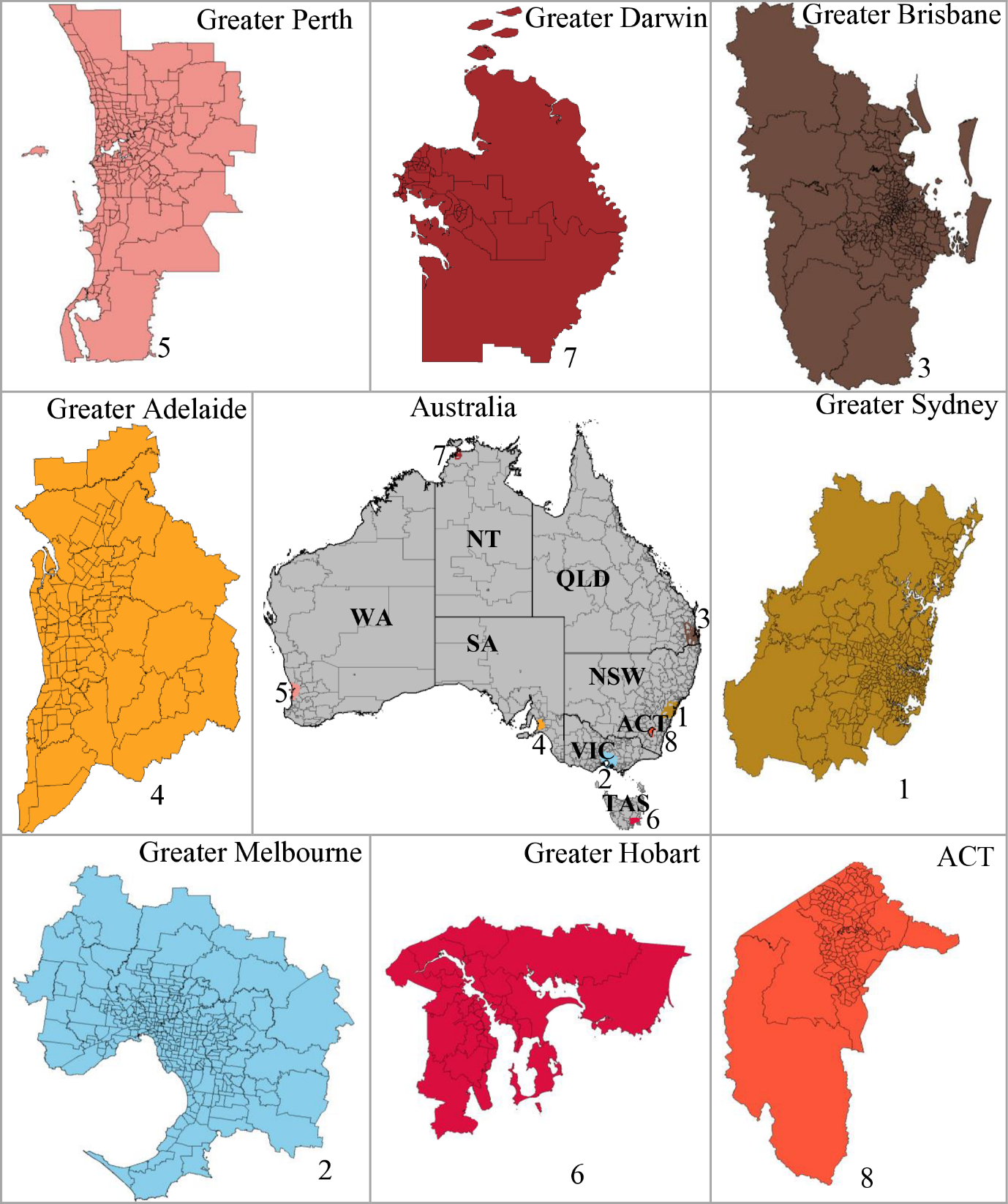


**Fig. S1: Study area map**

The Australian 2016 digital boundary sourced from the Australian Bureau of Statistics (ABS) was used to generate this map. The maps are partitioned by SA2 boundaries.


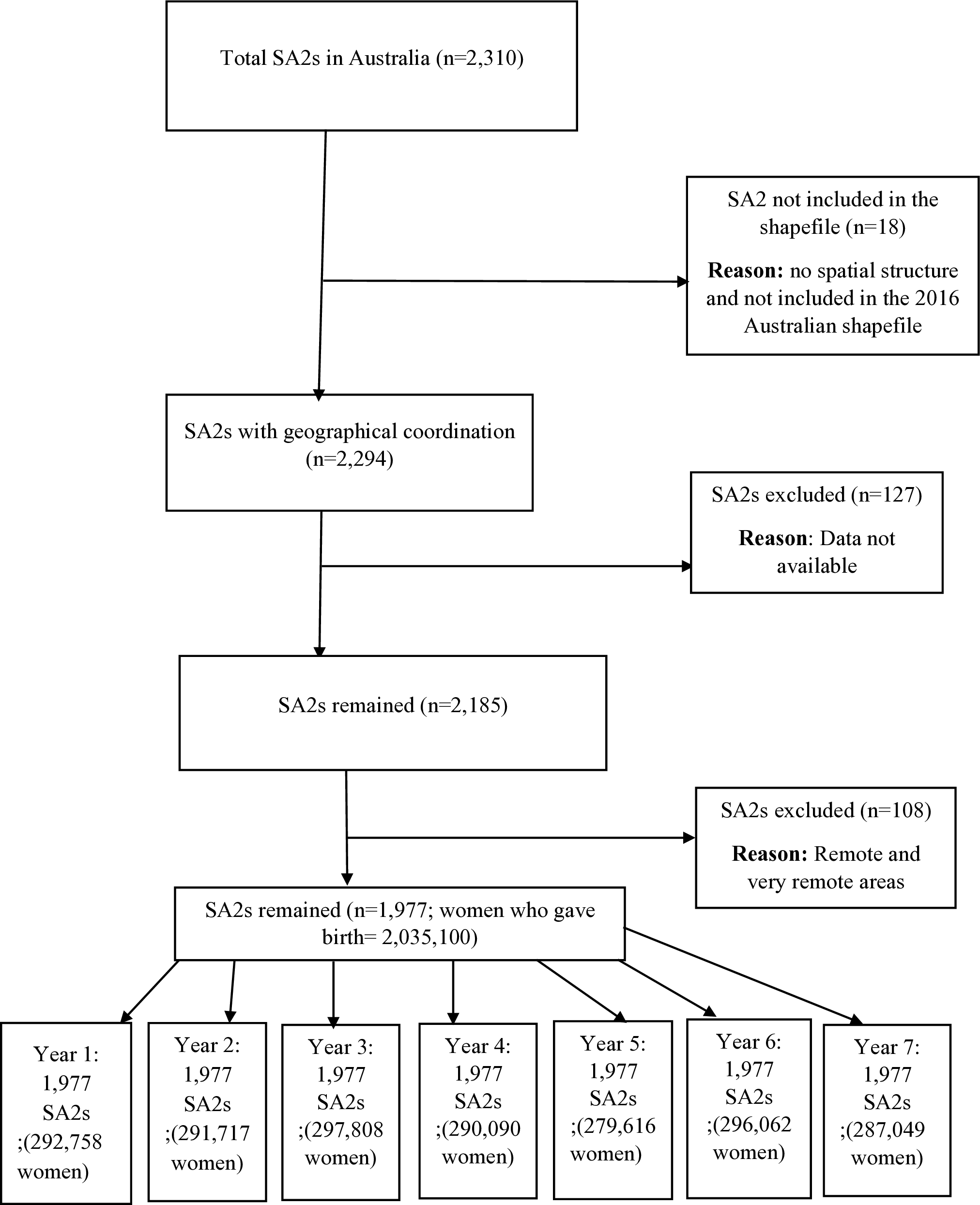


Fig. S2: A flow diagram illustrating the SA2 selection procedure


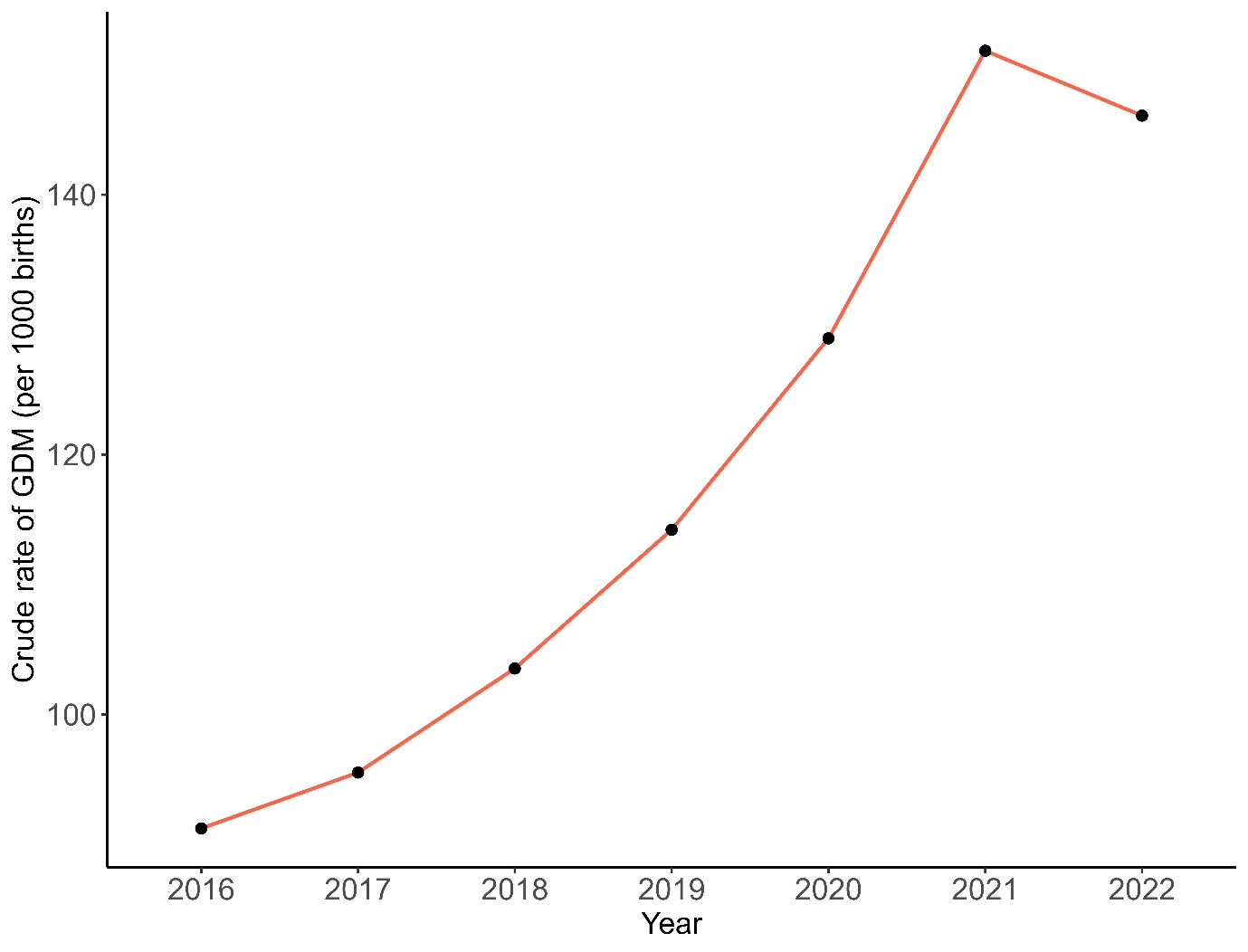


Fig. S3: Trends in the crude incidence of GDM between 2016 and 2022.

**Table S1: Measures of outcome and exploratory variables**

| **Variables** | **Definition** |
| --- | --- |
| **Outcome variables** |  |
| GDM^a^ | Annual count of GDM per SA2 between 2016 and 2022 |
| Women who gave birth^b^ | Annual count of women who gave birth between 2016 and 2022 |
| **Exploratory variables** |  |
| ***Social environment variables*** |  |
| Socioeconomic status^b^ | Socioeconomic indexes for areas (SEIFA) based on the 2016 Australian census-based Index of Relative Socio-economic Advantage and Disadvantage (IRSAD) [[1](#_ENREF_1)] |
| Ethnicity^b^ | Concentration of non-European migrant women per SA2 measured using location quotient (LQ) [[2](#_ENREF_2)] |
| ***Other sociodemographic variables*** |  |
| Age (continuous)^b^ | Women’s median age in SA2 |
| Population density^b^ | Number of people per square kilometre based on the 2016 ABS census report in SA2 |
| Urbanicity^b^ | SA2s were assigned to ‘major cities’ or ‘regional’ based on the 2016 ASGS [[3](#_ENREF_3)] |
| ***Natural environment*** |  |
| Greenness^c^ | Annual median residential greenness measured using Normalised Difference Vegetation Index (NDVI) in SA2 |
| Air pollution^d^ | Annual median concentration of PM_2.5_ and NO_2_ in SA2 |
| Temperature^c^ | Annual median land surface temperature (LST) in SA2 |

^a^NDSS

^b^ABS

^c^Landsat imagery, Google Earth Engine, Google

^d^Based on the national air pollution land-use regression model

**Text S2: Measurement of environmental greenness**

The most common metric of environmental greenness is the Normalised Differential Vegetation Index (NDVI). In Google Earth Engine (GEE), NDVI was calculated from *Landsat 8 Tier 1 Collection 2* surface reflectance imagery using the formula:

$$NDVI= \frac{NIR-Red}{NIR+Red}$$

where for Landsat 8, the Near-Infrared (NIR) band corresponds to Band 5 (B5), and Red band corresponds to Band 4 (B4). Surface reflectance, a unitless measure, quantifies the fraction of incoming solar radiation reflected by the Earth’s surface to the Landsat sensor, corrected for atmospheric effects. To reduce the noise and variabilities, a median pixel composting method was used across all scenes within each year (2016-2022). This method helps to remove clouds (high reflectance) and shadows (low reflectance). The 30m resolution grid data for each study year (2016-2022) were aggregated to SA2. Raster data was converted into a vector data layer in QGIS and aggregated to Statistical Area Level 2 (SA2) using the 2016 ASGS shapefile digital boundaries. Finally, the median NDVI values of all pixels within each SA2 were calculated for each year in QGIS. The detailed methodologies and the types of variables that were adjusted for are provided in previous work [[4](#_ENREF_4)]. The data were exported to Microsoft Excel and linked to outcome data at the SA2 level using the SA2 unique identifiers.

**Text S3: Measurement of environmental temperature**

*Landsat Tier 1 collection 2* surface reflectance satellite imagery method was used.[[5](#_ENREF_5)] *Landsat* is the preferred method for providing LST data at high spatial resolution, which helps aggregate data from the grid level to the SA2 level, compared to other satellite imageries (e.g., Moderate Resolution Imaging Spectroradiometer (MODIS), which provides 1 km spatial resolution data). The 2016 ABS digital boundary by SA2 was imported to GEE before aggregation from grids. Cloud masking was done to incorporate cloud-free and shadow-free pixels in the computation process. The LST estimation was adjusted for surface emissivity and greenness (measured by NDVI), similar to the previous studies [[6](#_ENREF_6)]. While Bands 4 and 5 were used to estimate the NDVI, Band 10 was used to compute the temperature brightness during LST retrieval, and a similar algorithm was followed in previous studies [[6](#_ENREF_6), [7](#_ENREF_7)]. The LST values for each calendar year from 2016 to 2022 were estimated by averaging the pixels in each SA2 as the temperature measures in the corresponding SA2s. As the default temperature unit in Landsat is Kelvin, it was first transformed to Celsius before estimating the median and mean values for each SA2 and year. Finally, the data was exported in Excel files and linked to the outcome variable dataset using the SA2 identifiers.

$$ԑ=0.004*(\frac{\left( NDVI-NDVImin \right)2}{NDVImax-NDVImin}+0.0986$$

LST=$\frac{Band10*0.1}{1+Band10*0.1*0.00115*\frac{ln(ԑ)}{1.438}}-273.15$

**Text S4: Generalised linear mixed-effect model (GLMM)**

Two mixed-effect models were fitted: Model 1 considered SA2 only as a random effect and covariates (annually measured natural and social environment factors) as fixed effects, while Model 2 extended Model 1 by adding year as a random effect. Random and fixed-effect variables can be assumed at both individual- and ecological-level studies, depending on the context. In our study, fixed effects are population-level independent variables assumed to have a constant influence on disease outcomes across geographical units. Whereas random effects account for unit-specific deviations and unobserved variability and correlation in the data, commonly associated with clustering variables, such as space and time, in our study.

We considered the denominator population as an offset variable in the model to account for variations in population size across SA2s [[8](#_ENREF_8)]. Models were compared using the Akaike information criterion (AIC), the deviance information criterion (DIC), and the marginalised log-likelihood. See the model specifications in Text S5 (p. 14) at the end of the supplementary material.

**Text S5****: Spatial autocorrelation test and neighbourhood matrix construction**

Neighbouring areas are assumed to share similar risk factors and to exhibit spatial dependence (autocorrelation). Spatial autocorrelation was examined for 1,977 SA2s using the regression residuals from the non-spatial models described above. Moran’s I spatial statistics for spatial autocorrelation was used, and a significant p-value suggested spatial autocorrelation [[9](#_ENREF_9)].

The neighbourhood weights matrix (W) was constructed using the queen contiguity method, an accurate spatial smoothing technique (an approach to reduce noise or data variability across areas) in Bayesian spatial analysis [[10](#_ENREF_10), [11](#_ENREF_11)]. The elements of ‘W’ are denoted as $Wij$ where $i$ and $j$ represent areas (SA2s). Each area (SA2) is assumed to have at least one neighbour with a common border, and adjacent areas are assumed to be correlated, whereas distal areas are not. $Wij$ follows a binary outcome, i.e. ‘0’ or ‘1’. $Wij$ is ‘1’ if SA2s are neighbours; otherwise, it was assigned ‘0’. With 1,977 SA2s across Australia, W constitutes a 1,977 X 1,977 matrix.

**Text S6: Spatiotemporal model specifications**

***Model 1: BYM-linear:***

*Y_it_* ∼ Poisson(*𝜆_it_*); *𝜆_it_* = *E_it_𝜌_it_*

log(*𝜌_it_*) = *𝜂_it_*

*𝜂it*= α + ∑ β*_i_*_,_*_t_*$x$*_i,t_* + u*_i_* + v*_i_* + (β+δi) × t*_i_* (1)

Where *Y_it_* represent the observed GDM count in each SA2 (*i=1,…1,977)* and year (*t=1,…,7)* [[12](#_ENREF_12)]; *𝜆_it_:* expected mean of the Poisson distribution for each SA2 and year*; 𝜌_it_:* relative risk for *i and t.* *E_it_* is the expected GDM count in each SA2(*i*) and year (*t*), calculated using indirect standardisation through the mean national incidence of GDM for each year, which serves as an offset in the modelling. α represents the intercept, ∑ β*_it_*$x$*_it_* indicates the covariates (fixed effects), including age, socioeconomic status, and environmental exposure measures (e.g., NO_2_). The structured (*u_i_*) and unstructured (v*_i_*) random effects are specified using *BMY2* model. β captures the global linear trend (overall GDM trend), and δ*i* the interaction between areas (SA2) and time (year) that explains the difference between the global trend (β) and the SA2-specific trend. The *u_i_* and *δ_i_* are modelled using the intrinsic conditional autoregressive (iCAR) distribution and independent and identically distributed (*iid)* models, respectively. For the unstructured random effects, random intercepts were specified for each spatial unit, and random slopes for each time were also allowed to vary across units.

***Model 2: BYM random walk 1(BYM-RW1):***

*𝜂it*= α + ∑ β*_i_*_,_*_t_*$x$*_i,t_* + u*_i_* + v*_i_* + γ*_t_* + ϕ*_t_* (2)

∑ β*_i_*_,_*_t_*$x$*_i,t_,* u*_i_*, v*_i_* are similar to the previous formula (1); γ*_t_* represents a temporally structured effect; ϕ*_t_* was specified by means of the exchangeable prior.

Due to the lack of prior knowledge, we used the default INLA non-informative priors: logGamma (mean=1 and precision=0.00005) for the random effects, and Normal (mean=0 and precision=0.001) for the fixed effects.

**Table S2: Summary of sociodemographic characteristics of women who gave birth in Australia over time, 2016-222 (N=1,977 SA2s).**

| **Sociodemographic**  **characteristics** | **Year** | | | | | | |
| --- | --- | --- | --- | --- | --- | --- | --- |
|  | **2016** | **2017** | **2018** | **2019** | **2020** | **2021** | **2022** |
| **Age, Median (IQR)** | 31.0 (16.4, 37.2) | 31.2 (16.4, 36.0) | 31.3 (16.2, 36.8) | 31.5 (16.4, 36.4) | 31.5 (16.1, 36.5) | 31.7 (16.3, 37.3) | 31.9 (16.2, 37.1) |
| **Age group, N (%)** |  |  |  |  |  |  |  |
| 15-19 | 6,933 (2.4) | 6,827 (2.3) | 6,381(2.1) | 5,980 (2.1) | 5,461(1.9) | 4,983 (1.7) | 4,938 (1.7) |
| 20-24 | 33,639 (11.4) | 32,906 (11.2) | 32,907(11.0) | 31,079 (10.7) | 28,616 (10.2) | 28,078 (9.4) | 26,647 (9.2) |
| 25-29 | 78,282 (26.6) | 77,673 (26.5) | 78,440 (26.2) | 75,157 (25.8) | 71,037 (25.3) | 74,256 (24.9) | 70,921(24.6) |
| 30-34 | 106,017 (36.1) | 104,960 (35.8) | 107,582 (36.0) | 105,514 (36.2) | 103,138 (36.7) | 111,488 (37.5) | 107,158 (37.2) |
| 35-39 | 55,376 (18.8) | 56,909 (19.4) | 59,608 (19.9) | 59,715 (20.5) | 59,040 (21.0) | 64,392 (21.7) | 63,913 (22.2) |
| ≥40 | 13,585 (4.6) | 13,518 (4.6) | 14,161(4.7) | 13,721 (4.7) | 13,527 (4.8) | 14,053 (4.7) | 14,729 (5.2) |
| **Ethnicity, N (%)** |  |  |  |  |  |  |  |
| Australia | 185,097 (62.5) | 183,501 (62.2) | 187,900 (62.4) | 182,423 (62.2) | 176,411 (62.3) | 189,422 (63.2) | 185,562 (63.8) |
| Oceania (excludes Australia) | 11,818 (4.0) | 11,812 (4.0) | 11,658 (3.9) | 11,318 (3.9) | 10,483 (3.7) | 10,520 (3.5) | 10,285 (3.5) |
| Americas | 5,460 (1.8) | 5,716 (1.9) | 6,128 (2.0) | 6,018 (2.1) | 5,826 (2.1) | 6,689 (2.2) | 6,287 (2.2) |
| North Africa and the Middle East | 9,450 (3.2) | 9,651 (3.3) | 10,228 (3.4) | 9,130 (3.1) | 8,711 (3.1) | 8,772 (2.9) | 8,555 (2.9) |
| North-East Asia | 16,724 (5.6) | 16,408 (5.6) | 15,446 (5.1) | 15,309 (5.2) | 13,459 (4.8) | 12,819 (4.3) | 11,827 (41.1) |
| Northwest Europe | 12,845 (4.3) | 12,323 (4.2) | 12,325 (4.1) | 12,028 (4.1) | 11,555 (4.1) | 12,984 (4.3) | 11,724 (4.0) |
| South and Central Asia | 24,625 (8.3) | 26,031 (8.8) | 27,449 (9.1) | 27,891 (9.5) | 28,279 (10.0) | 29,783 (9.9) | 28,960 (10.0) |
| South-East Asia | 17,382 (5.9) | 16,864 (5.7) | 17,160 (5.7) | 16,833 (5.7) | 16,120 (5.7) | 16,013 (5.3) | 15,349 (5.3) |
| South-East Europe | 5,471 (1.8) | 5,478 (1.9) | 5,446 (1.8) | 5,249 (1.8) | 5,086 (1.8) | 5,268 (1.8) | 4,869 (1.7) |
| Sub-Saharan Africa | 7,152 (2.4) | 7,015 (2.4) | 7,494 (2.5) | 7,167 (2.4) | 7,161 (2.5) | 7,148 (2.4) | 7,263 (4.5) |
| **Concentration of migrants** |  |  |  |  |  |  |  |
| Low (LQ≤1) | 175,915 (60.1) | 172,655 (59.2) | 176,053 (59.1) | 170,714 (58.8) | 165,723 (59.3) | 174,753 (59.0) | 168,928 (58.8) |
| High (LQ>1) | 116,843 (39.9) | 119,062 (40.8) | 121,755 (40.8) | 119,376 (41.2) | 113,893 (40.7) | 121,309 (41.0) | 118,121 (41.2) |
| **Socioeconomic status, N (%)** |  |  |  |  |  |  |  |
| Q1 (most disadvantaged) | 52,332 (17.9) | 52,975 (18.2) | 54,023 (18.1) | 52,025 (17.9) | 49,490 (17.7) | 50,436 (17.0) | 50,031 (17.4) |
| Q2 | 52,744 (18.0) | 52,815 (18.1) | 55,240 (18.5) | 53,124 (18.3) | 51,775 (18.3) | 54,262 (18.3) | 54,270 (18.9) |
| Q3 | 64,128 (21.9) | 63,922 (21.9) | 65,037 (21.8) | 64,629 (22.3) | 62,693 (22.4) | 67,432 (22.8) | 65,911 (22.9) |
| Q4 | 63,373 (21.6) | 62,584 (21.5) | 63,613 (21.4) | 62,886 (21.7) | 60,694 (22.7) | 65,358 (22.1) | 63,014 (21.9) |
| Q5 (least disadvantaged) | 60,181 (20.1) | 59,421 (20.4) | 59,895 (20.1) | 57,426 (19.7) | 54,964 (19.7) | 58,574 (19.8) | 53,823 (18.8) |
| **Remoteness, N (%)** |  |  |  |  |  |  |  |
| Major cities | 2,210,00 (75.5) | 2,200,32 (75.4) | 2,236,84 (75.1) | 2,179,40 (75.1) | 208,601 (74.6) | 2,198,84 (74.3) | 2,115,32 (73.7) |
| Regional | 71,758 (24.5) | 71,685 (24.6) | 74,124 (24.9) | 72,150 (24.9) | 710,15 (25.4) | 76,178 (25.7) | 75,517 (26.3) |

**Data sources:**

*The number of women who gave birth, aggregated by SA2 and years, was obtained from ABS.*

*Remoteness and socioeconomic status classification were based on the 2016 ABS census data.*

**Women who gave birth include those with GDM.*

*LQ: location quotient; Q: quintile; IQR: interquartile range*

**Table S3: Summary of sociodemographic characteristics of women with GDM in Australia over time, 2016-2022 (N=1,977 SA2s).**

| **Sociodemographic**  **characteristics** | **Year** | | | | | | |
| --- | --- | --- | --- | --- | --- | --- | --- |
|  | **2016** | **2017** | **2018** | **2019** | **2020** | **2021** | **2022** |
| **Age, Median (IQR)** | 33.0 (16.1, 44.4) | 33.3 (16.4,45.3) | 33.0 (15.2, 46.0) | 34.1 (14.2, 47.6) | 33.5 (15.8, 48.2) | 34.9 (15.0, 49.2) | 34.8 (16.1, 49.6) |
| **Age group, N (%)** |  |  |  |  |  |  |  |
| 15-19 | 95 (0.4) | 107 (0.4) | 115 (0.4) | 101 (0.3) | 96 (0.3) | 118 (0.3) | 98 (0.2) |
| 20-24 | 1,356 (5.1) | 1,376 (5.0) | 1,520 (5.0) | 1,618 (4.9) | 1,643 (4.6) | 1,872 (4.2) | 1,838 (4.1) |
| 25-29 | 4,558 (17.2) | 4,690 (17.0) | 5,272 (17.3) | 5,362 (16.4) | 5,937 (16.7) | 7,366 (16.7) | 7,603 (17.0) |
| 30-34 | 9,675 (36.5) | 9,664 (35.0) | 10,781 (35.3) | 11,548 (35.3) | 12,769 (35.9) | 15,909 (36.1) | 15,919 (35.6) |
| 35-39 | 8,101 (30.6) | 8,742 (31.7) | 9,528 (31.2) | 10,395 (31.7) | 11,219 (31.6) | 14,108 (32.0) | 14,268 (31.9) |
| ≥40 | 2,687 (10.2) | 2,995 (10.9) | 3,313 (10.9) | 3,728 (11.4) | 3,868 (10.9) | 4,694 (10.7) | 4,949 (11.1) |
| **Ethnicity, N (%)** |  |  |  |  |  |  |  |
| Australia | 14,397 (52.1) | 14,711 (51.1) | 16,043 (50.5) | 17,158 (50.0) | 9,876 (26.4) | 18,135 (39.4) | 27,635 (60.4) |
| Oceania (excludes Australia) | 1,068 (3.9) | 1,046 (3.6) | 1,137 (3.6) | 1,292 (3.8) | 1,417 (3.8) | 1,354 (2.9) | 1,354 (3.0) |
| Americas | 462 (1.7) | 473 (1.6) | 499 (1.6) | 556 (1.6) | 425 (1.1) | 549 (1.2) | 667 (1.5) |
| North Africa and the Middle East | 1,516 (5.5) | 1,703 (5.9) | 2,018 (6.3) | 2,095 (6.1) | 2,192 (5.8) | 2,542 (5.5) | 1,192 (2.6) |
| North-East Asia | 2,251 (8.1) | 2,386 (8.3) | 2,454 (7.7) | 2,776 (8.1) | 2,643 (7.1) | 2,918 (6.3) | 2,290 (5.0) |
| Northwest Europe | 1,208 (4.4) | 1,092 (3.8) | 1,228 (3.9) | 1,135 (3.3) | 923 (2.5) | 1,096 (2.4) | 1,119 (2.4) |
| South and Central Asia | 3,480 (12.6) | 4,217 (14.6) | 4,741 (14.9) | 5,379 (15.7) | 5,706 (15.2) | 6,342 (13.8) | 6,728 (14.7) |
| South-East Asia | 1,279 (4.6) | 1,437 (5.0) | 1,752 (5.5) | 1,986 (5.8) | 2,149 (5.7) | 2,615 (5.7) | 3,365 (7.4) |
| South-East Europe | 444 (1.6) | 474 (1.6) | 524 (1.6) | 533 (1.6) | 546 (1.5) | 627 (1.4) | 423 (0.9) |
| Sub-Saharan Africa | 601 (2.2) | 649 (2.3) | 759 (2.4) | 846 (2.5) | 948 (2.5) | 1,089 (2.4) | 946 (2.1) |
| Unspecified | 916 (3.3) | 612 (2.1) | 628 (2.0) | 575 (1.7) | 10,648 (28.4) | 8,818 (19.1) | 27 (0.1) |
| **Concentration of migrants, N (%)** |  |  |  |  |  |  |  |
| Low | 14,724 (55.1) | 14,906 (52.3) | 16,120 (51.1) | 16,945 (51.5) | 18,585 (51.5) | 23,020 (51.5) | 21,235 (50.1) |
| High | 11,984 (44.9) | 12,962 (47.7) | 14,715 (48.9) | 16,188 (48.5) | 17,470 (48.5) | 21,710 (48.5) | 20,700 (49.4) |
| **Socioeconomic status, N (%)** |  |  |  |  |  |  |  |
| Q1 (most disadvantaged) | 5,507 (20.6) | 5,692 (20.4) | 6,327 (20.5) | 6,770 (20.4) | 7,345 (20.4) | 8,696 (19.4) | 6,901 (16.5) |
| Q2 | 5,053 (18.9) | 5,343 (19.1) | 5,896 (19.1) | 6,352 (19.2) | 7,018 (19.5) | 8,681 (19.4) | 7,538 (17.9) |
| Q3 | 5,516 (20.7) | 5,884 (21.1) | 6,392 (20.7) | 6,948 (20.9) | 7,920 (21.9) | 9,749 (21.7) | 9,870 (23.5) |
| Q4 | 5,852 (21.9) | 6,028 (21.6) | 6,788 (22.0) | 7,418 (22.4) | 8,008 (22.2) | 10,159 (22.7) | 10,173 (24.3) |
| Q5 (least disadvantaged) | 4,780 (17.9) | 4,921 (17.7) | 5,432 (17.6) | 5,645 (17.1) | 5,764 (15.9) | 7,445 (16.6) | 7,453 (17.8) |
| **Remoteness, N (%)** |  |  |  |  |  |  |  |
| Major cities | 20,592 (77.1) | 21,635 (77.6) | 23,958 (77.7) | 25,674 (77.5) | 27,807 (77.1) | 34,199 (76.5) | 31,353 (74.8) |
| Regional | 6,116 (22.9) | 6,233 (22.4) | 6,877 (22.3) | 7,459 (22.5) | 8,248 (22.9) | 10,531 (23.5) | 10,582 (25.2) |

**Data sources:**

*The number of women with GDM, aggregated by SA2 and year, was obtained from the NDSS.*

*Remoteness and socioeconomic status classification were based on the 2016 ABS census data.*

*LQ: location quotient; Q: quintile; IQR: interquartile range*


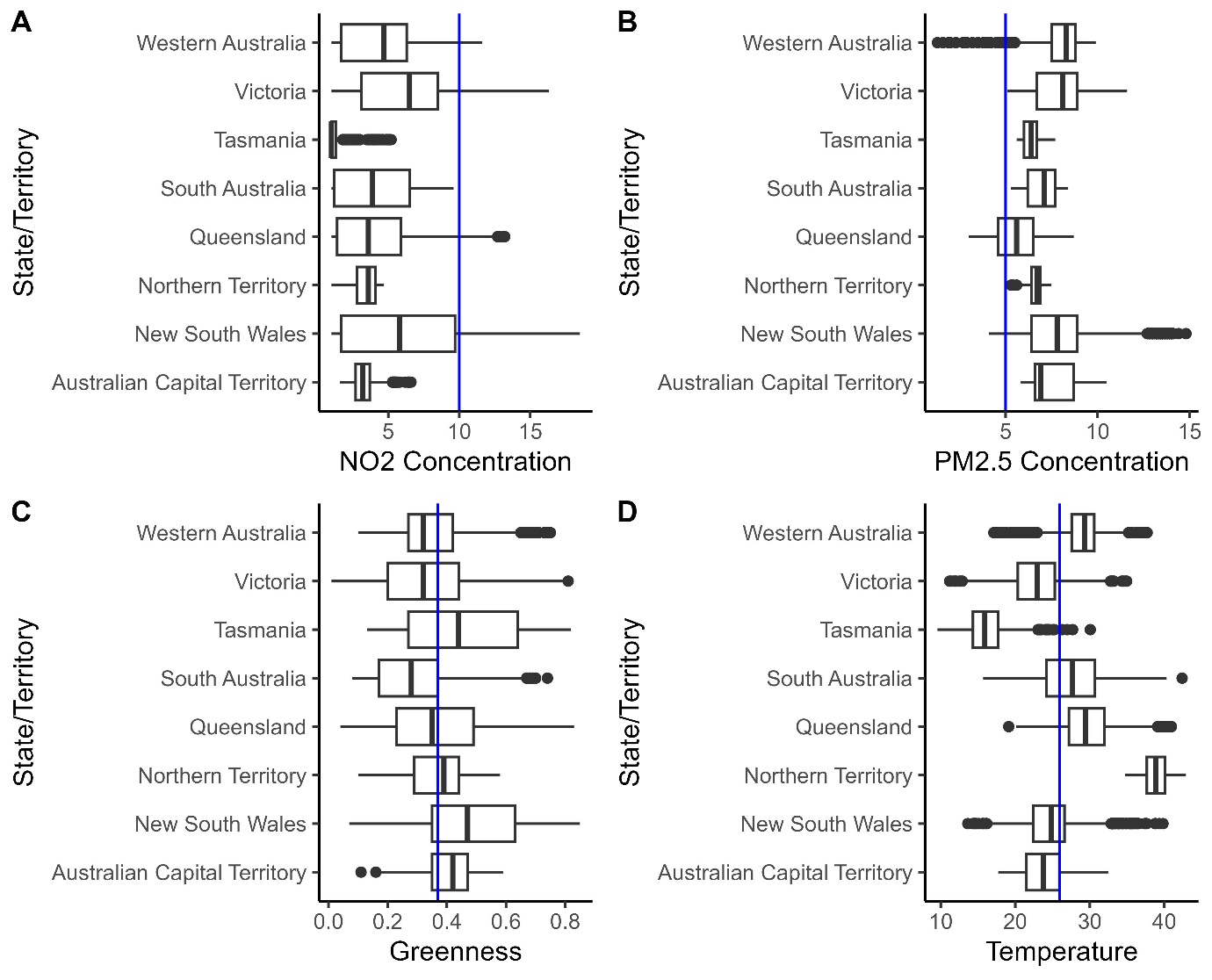


**Fig. S4: Disparities in the natural environment characteristics by states/territories.**

*We averaged the yearly SA2-level natural environment measures to obtain the overall estimate across states/territories. Annual median NO_2_ pollution (ppb) (A), PM_2.5_ pollution (µg/m3) (B), greenness (NDVI) (C), and temperature (°C) (D) by state/territory, Australia, 2016-2022. The blue vertical line for NO_2_ and PM_2.5_ indicates the maximum thresholds set by the World Health Organisation (WHO) in 2021. For greenness and temperature, the blue vertical line indicates the median estimates of each environmental characteristic.*

**Text S7: Disparities in natural environment attributes across major cities**

Disproportionate exposure to natural environment attributes was also observed across cities. The highest and the lowest NO2 concentrations were recorded in Sydney and Hobart, respectively (Fig. S5a). Surprisingly, all the cities recorded air pollution (PM_2.5_) exceeding the 2021 WHO annual PM_2.5_ recommendation threshold (>5µg/m^3^). PM_2.5_ concentration in Sydney, Melbourne, and Perth also exceeded the national median concentration (Fig. S5b). Moreover, residential greenness also significantly varies between and within cities. The majority of neighbourhoods in Perth (74.3%) and Adelaide (73.5%) were less green compared with other cities (Fig. S5c). All areas of Darwin recorded temperatures above the national average, whereas all areas of Hobart recorded temperatures below the average (Fig. S5d).


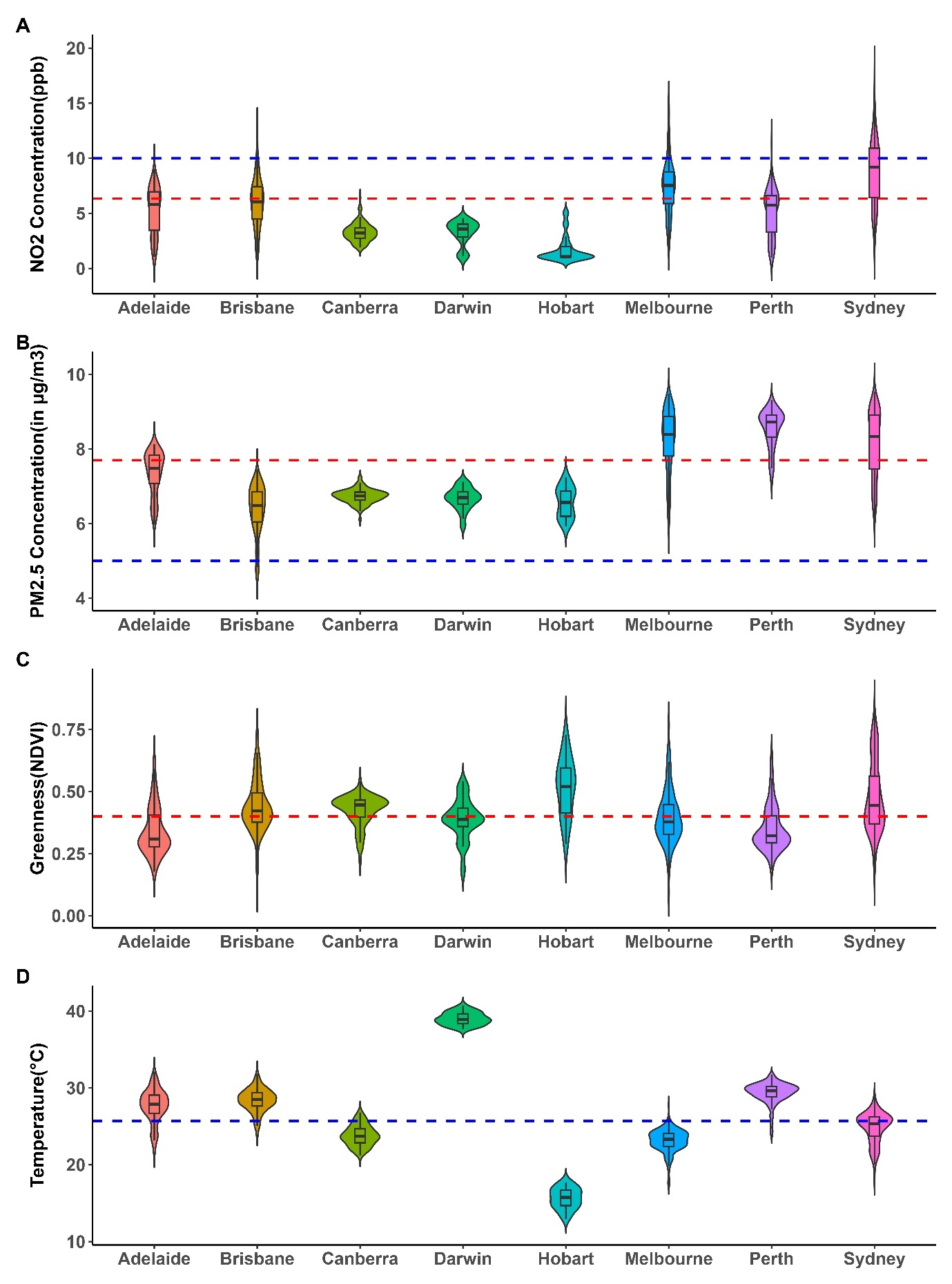


**Fig. S5: Violin plots illustrating the disparities in natural environment characteristics by major cities.**

*We averaged the yearly SA2-level natural environment measures to obtain the overall estimate across cities. Annual median NO_2_ pollution (ppb) (A), PM_2.5_ pollution (µg/m^3^) (B), greenness (NDVI) (C), and temperature (°C) (D) by city, Australia, 2016-2022. The blue vertical lines for NO_2_ and PM_2.5_ indicate the maximum thresholds set by the WHO in 2021. For greenness and temperature, the blue vertical line indicates the median estimates of each environmental characteristic.*


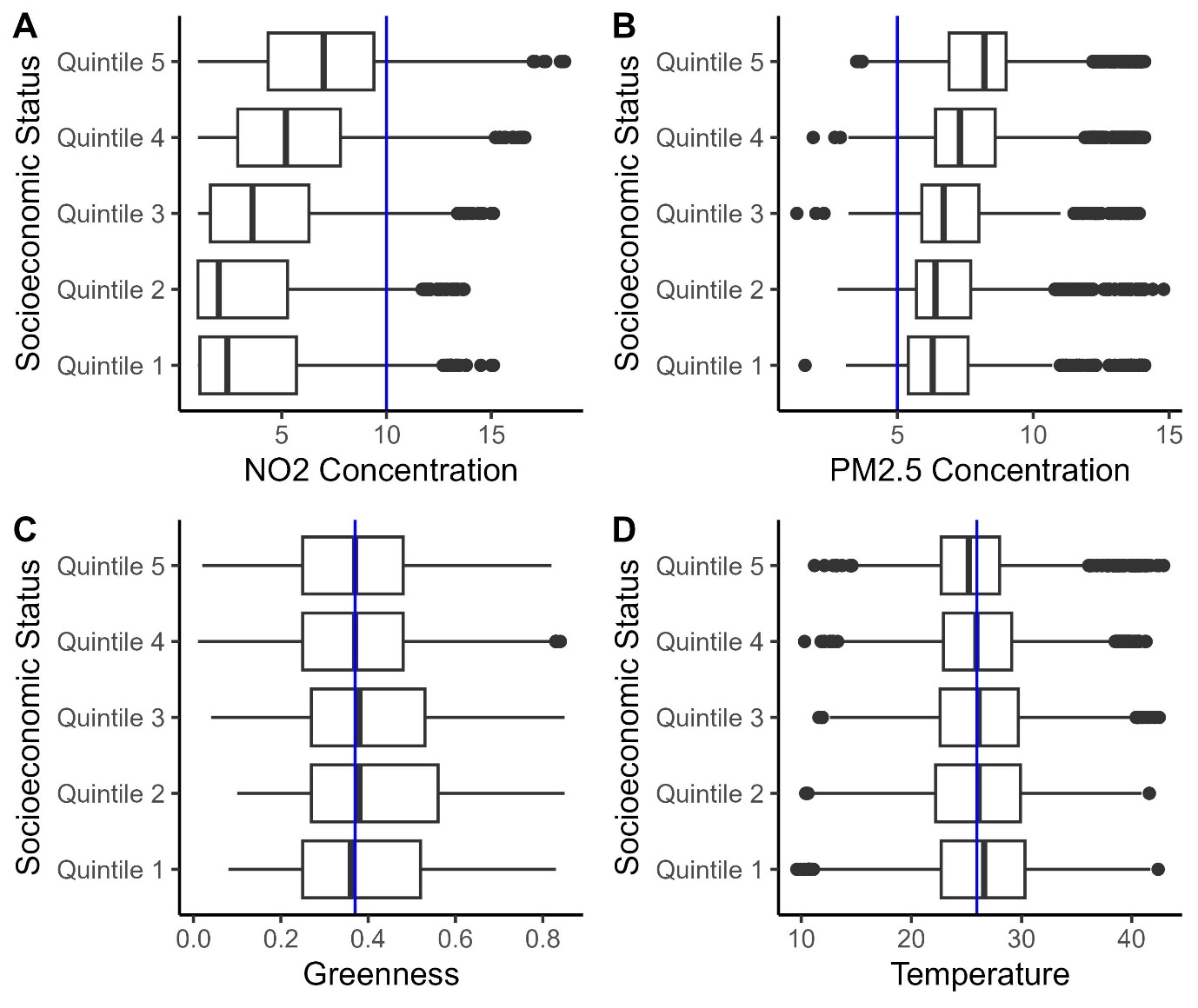


Fig. S6: Disparities in natural environment characteristics by socioeconomic status.

*We averaged the yearly SA2-level natural environment measures to obtain the overall estimate across socioeconomic groups. Annual median NO_2_ pollution (ppb) (A), PM_2.5_ pollution (µg/m^3^) (B), greenness (NDVI) (C), and temperature (°C) (D) by state/territory, Australia, 2016-2022. The blue vertical lines for NO_2_ and PM_2.5_ indicate the maximum thresholds set by the WHO in 2021. For greenness and temperature, the blue vertical line indicates the median estimates of each environmental characteristic. While ‘quintile 1’ represents the most socioeconomically disadvantaged, ‘quintile 5’ is the least disadvantaged.*

**Geographical distribution of natural environment measures**

***NO_2_ concentration***

During 2016 and 2022, the inner regions of capital cities, namely Sydney (New South Wales), Melbourne (the state of Victoria), Brisbane (Queensland), Perth (Western Australia), and Adelaide (South Australia), were the most polluted areas. The significant inner areas of Sydney, followed by Melbourne, exceeded the World Health Organisation’s NO_2_ pollution guideline (10 ppb). While very few areas of Canberra (Australian Capital Territory) and Hobart (Tasmania) were moderately affected over time, the entire Darwin (Northern Territory) was mildly affected, with a median concentration below 5. All regions of the rest of the country were moderately affected, far below the WHO’s recommendation (Fig. S7).
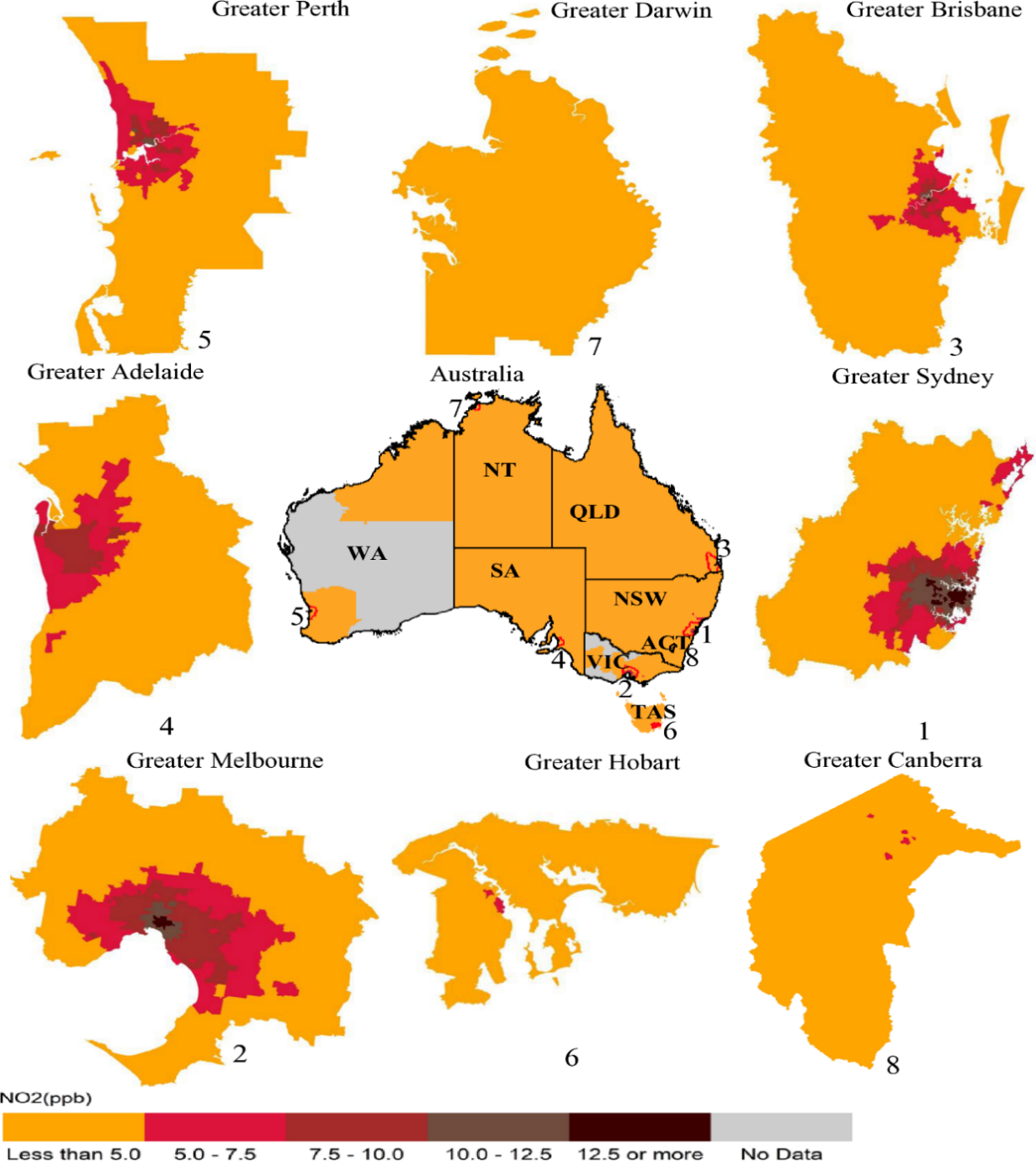


Fig. S7: Geographical distribution of air pollution (NO_2_) at the SA2 level in Australia over 2016-2022.

*NSW: New South Wales, VIC: Victoria; QLD: Queensland; WA: Western Australia; SA: South Australia; NT: Northern Territory; ACT: Australian Capital Territory; TAS: Tasmania.*

***PM_2.5_ concentration***

Based on the seven-year annual average PM_2.5_ concentration, all areas of the country were significantly polluted, exceeding the WHO’s 2021 annual air quality recommendation of below 5 µg/m³ of PM_2.5_ concentration. The inner areas of almost all capital cities recorded high air pollution, with very high pollution levels in the majority of the three major cities: Sydney, Melbourne, and Perth, which exceeded 10 µg/m³, twice the WHO’s recommended limit (Fig. S8).


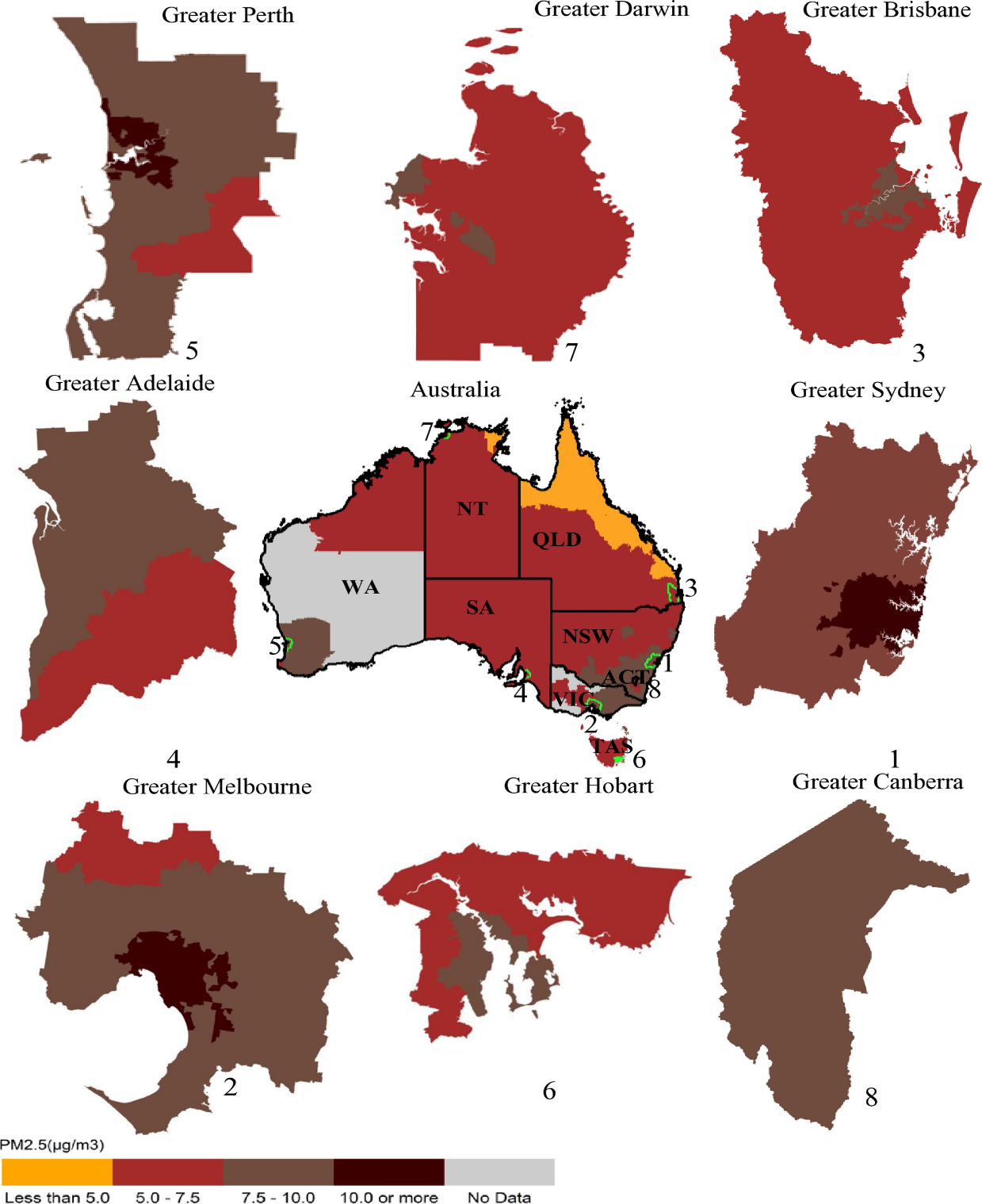


Fig. S8: Geographical distribution of air pollution (PM_2.5_) at the SA2 level in Australia over 2016-2022.

*NSW: New South Wales, VIC: Victoria; QLD: Queensland; WA: Western Australia; SA: South Australia; NT: Northern Territory; ACT: Australian Capital Territory; TAS: Tasmania.*

***Geographical distribution of greenness***

Fig. S9 illustrates the geographical distribution of residential greenness, which varies across small areas and cities, and states/territories in Australia. In all cities, the inner areas were the least green compared with the outer. Notably, residential greenness was low in significant areas of Perth, Adelaide, and Melbourne.


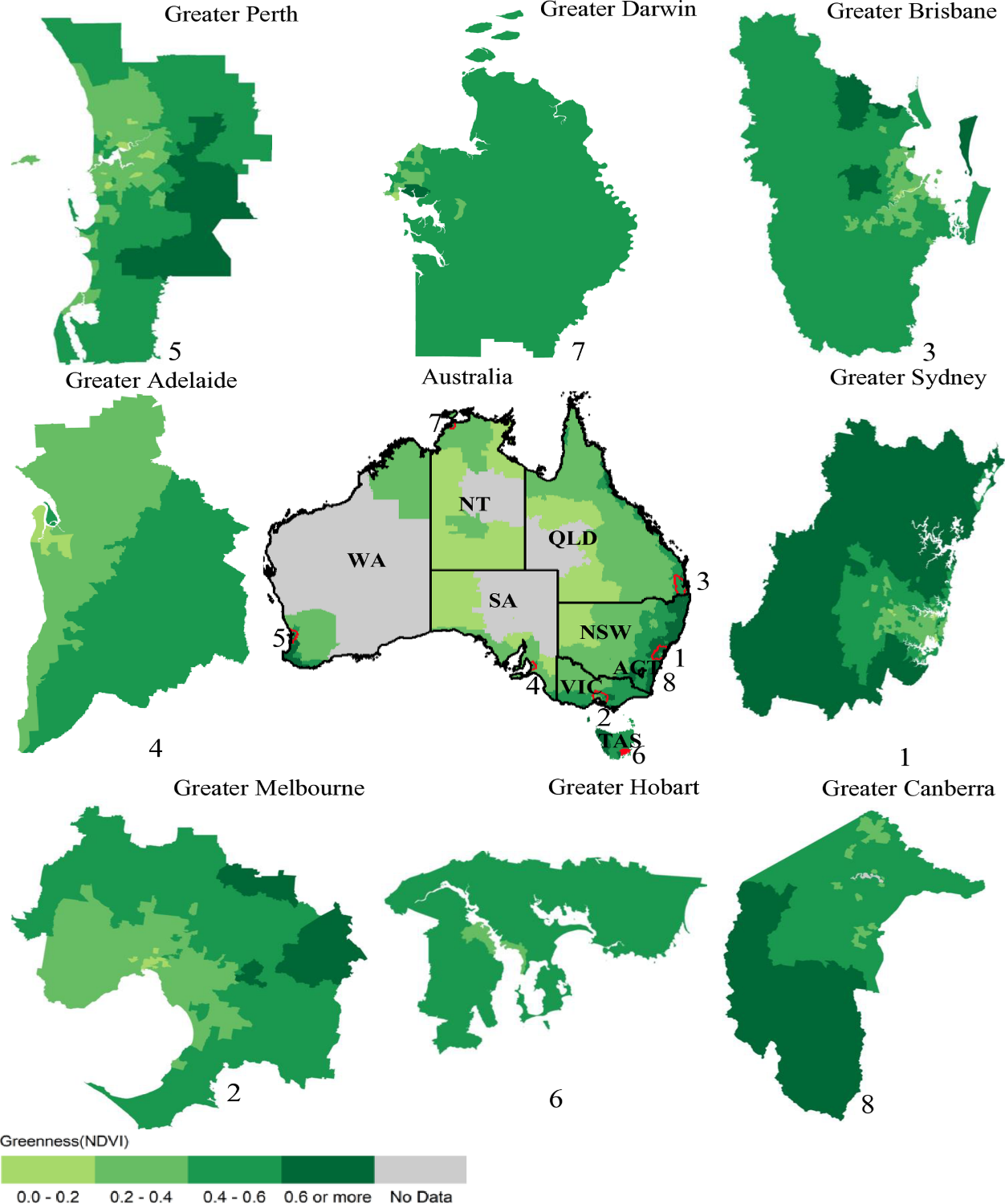


Fig. S9: Geographical distribution of residential greenness (based on NDVI) at the SA2 level in Australia over 2016-2022.

*NSW: New South Wales, VIC: Victoria; QLD: Queensland; WA: Western Australia; SA: South Australia; NT: Northern Territory; ACT: Australian Capital Territory; TAS: Tasmania.*

***Geographical distribution of temperature***

Fig. S10 show the geographical distribution of temperature at the SA2 level across states/territories and capital cities in Australia. Based on the median annual temperature estimate, variations in temperature exposure were observed between SA2s and cities in Australia over time. While Darwin (Northern Territory) was the hottest city (≥29.82 °C), Hobart (Tasmania) was the coolest (23.3 °C). Outer areas in Sydney and Melbourne were cooler than the inner. Significant areas of Perth (Western Australia) were hot.
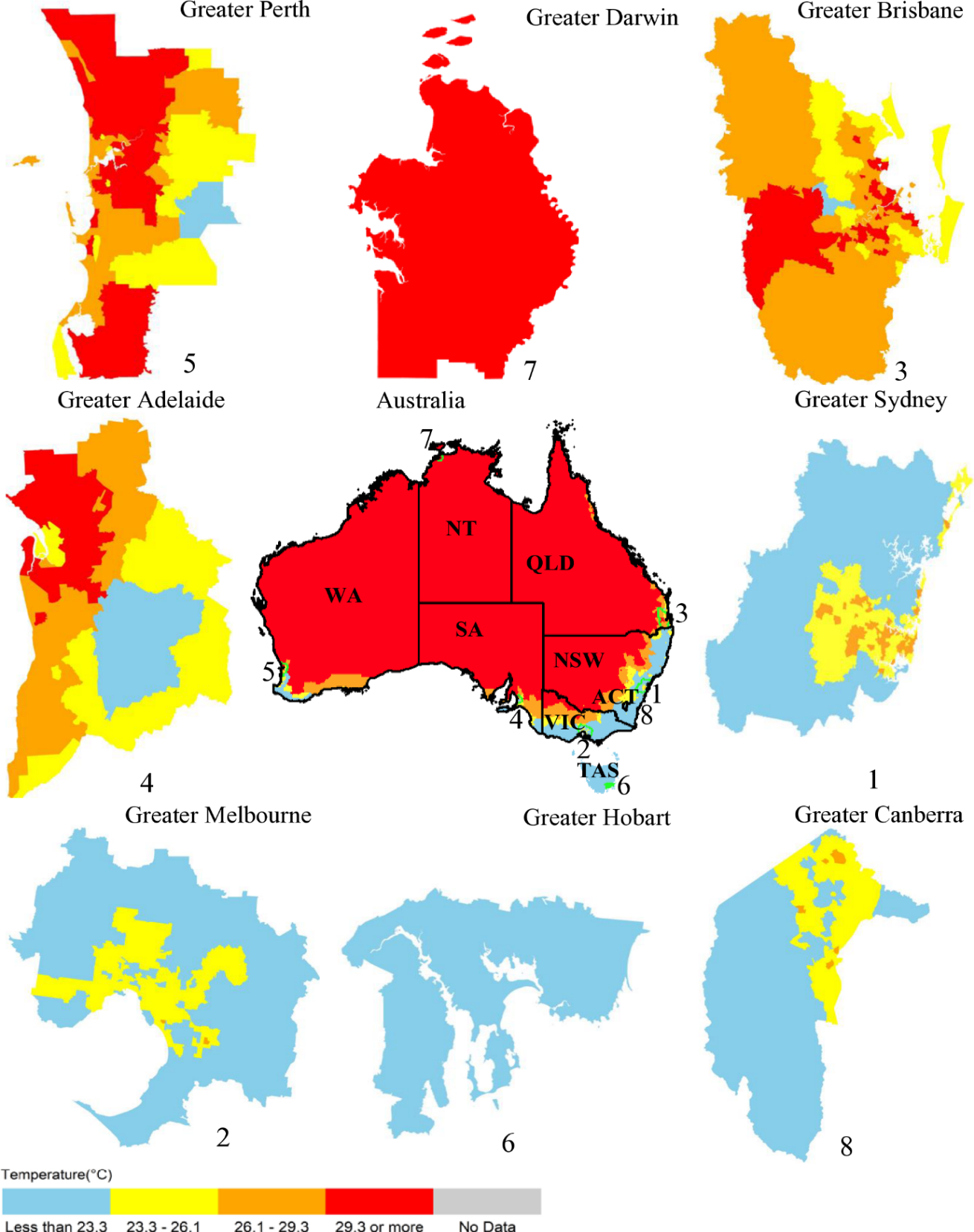


Fig. S10: Geographical distribution of ambient temperature at the SA2 level in Australia over 2016-2022.

*NSW: New South Wales, VIC: Victoria; QLD: Queensland; WA: Western Australia; SA: South Australia; NT: Northern Territory; ACT: Australian Capital Territory; TAS: Tasmania.*

**Table S4: Summary of Spearman correlation between natural environment measures.**

|  | NO_2_ concentration | Median PM_2.5_ concentration | Residential greenness (Normalized Difference Vegetation Index [NDVI]) | Temperature (LST) |
| --- | --- | --- | --- | --- |
| NO_2_ concentration | 1.00 |  |  |  |
| PM_2.5_ concentration | 0.78* | 1.00 |  |  |
| Residential greenness (Normalised Difference Vegetation Index [NDVI]) | -0.39 | -0.18 | 1.00 |  |
| Temperature (LST) | -0.04 | 0.28 | -0.36 | 1.00 |

NO_2_ and PM_2.5_ concentrations strongly correlate (correlation coefficient=0.78), and the two variables were modelled separately. All measures use median at SA2.

*Strong correlation

**Table S5: Non-spatial models (generalised linear mixed-effect models [GLMMs]).**

|  | **AIC** | **DIC** | **Log-likelihood** | **Description** |
| --- | --- | --- | --- | --- |
| **Models** |  |  |  |  |
|  | | | | |
| Model 1 | 87998 | 87969 | -43985 | A model incorporating a single pollutant (NO_2_), temperature, and other sociodemographic covariates as fixed effects. Area-only (SA2) was considered as a random effect. |
| **Model 2** | **87638** | **87607** | **-43804** | Model 1 plus time (year) with a random effect. |

**Table S6: Summary of the non-spatial modelling on the association of natural environment and GDM risk (estimates from Model 2, Table S5).**

| **Natural environment attributes** | **Adjusted Risk Ratio (ARR) (credible intervals [CrIs])** |
| --- | --- |
| PM_2.5_ concentration | 1.00 (0.99, 1.00) |
| NO_2_ concentration | 0.99 (0.98, 1.00) |
| Residential greenness (a 0.10 increase in NDVI) | **0.82(0.76, 0.83) *** |
| Temperature (LST) | 0.99 (0.99, 1.00) |

*The estimates are from single pollutant models.*

**Table S7: Spatiotemporal ecological model comparison, 2016-2022.**

|  | **AIC** | **DIC** | **Log-likelihood** | **Description** |
| --- | --- | --- | --- | --- |
| **Model 3a (BYM-linear)** | **85058** | **84419** | **-42908** | This assumes a parametric dynamic trend, with linear time trends in each area (SA2).[[13](#_ENREF_13), [14](#_ENREF_14)] This model allows for estimating the overall risk trends and specific trends across SA2. This is a single-pollutant (NO_2_) and temperature model adjusted for other covariates, such as neighbourhood socioeconomic status. |
| **Model 3b (BYM-linear)** | **85065** | **84428** | **-42914** | Model 3a (excluding NO_2_) + PM_2.5_ |
| Model 4 (BYM-RW1) | 85345 | 84927 | -43014 | This model extends Model 3a by adding a ‘random walk of order 1 (RW1)’ to account for the non-parametric dynamic evolution across areas over time.[[13-15](#_ENREF_13)] |

Note: The interpretation of the association between greenness and temperature and GDM risk was based on estimates of Model 3a, as the model estimates indicate the best-fitted model. The association between NO_2_ and GDM risk was interpreted based on Model 3a. Whereas estimates (Adjusted risk ratio [ARR] with CrIs from Model 3b were used to interpret the association between PM2.5 and GDM risk.

**Table S8: A table comparing the BYM-based Queen contiguity (binary) and row-standardised weights.**

| **Models** | **WAIC** | **DIC** | **Log Score (conditional predictive ordinate [CPO])** | **Comment** |
| --- | --- | --- | --- | --- |
| Binary adjacency (Model: ‘BYM2-linear’) | **85062.1** | **84423.5** | **42660.7** | The preferred model |
| Row-standardised (Model: ‘generic1’) | 85419.5 | 85068.6 | 42676.8 |  |

*WAIC: Watanabe-Akaike Information Criterion; DIC: Deviance Information Criterion.*

**Table S9: Sensitivity analysis by categorical exposures (air pollution measures and greenness).**

| **Natural environment attributes** | **Adjusted Risk Ratio (ARR) (CrIs)** |
| --- | --- |
| **Residential greenness (Normalised Difference Vegetation Index [NDVI])** |  |
| Low (<0.2) | Ref |
| Moderate (0.2-0.6) | **0.94 (0.94, 0.96)*** |
| High (≥0.6) | **0.86 (0.83, 0.90)*** |
| **PM_2.5_ concentration** |  |
| Low (≤5 µg/m^3^) | Ref |
| High (>5 µg/m^3^) | 0.95(0.91, 1.00) |
| **NO_2_ concentration** |  |
| Low (≤10 ppb) | Ref |
| High (>10 ppb) | 1.02 (0.97, 1.06) |

*The model was adjusted for median age, concentration of migrant women (high vs low concentration), population density, and neighbourhood socioeconomic status. Ref: reference category. ppb: denotes parts per billion. NDVI: Normalised difference vegetation index. *denotes significant association.*

**Table S10: Sensitivity analysis showing the association between natural environment and GDM, excluding data during the COVID-19 pandemic (2020-2021).**

| **Natural environment attributes** | **Adjusted Risk Ratio (ARR) (CrIs)** |
| --- | --- |
| PM_2.5_ concentration | 0.99 (0.98, 1.00) |
| NO_2_ concentration | 1.00 (0.99, 1.01) |
| Residential greenness (Normalised Difference Vegetation Index [NDVI]) | **0.62 (0.57, 0.68) *** |
| Temperature (LST) | 0.99 (0.98, 1.00) |

*The estimates (ARRs with corresponding CrIs) for NO_2_ and PM_2.5_ were extracted from the single-pollutant models fitted separately due to the high correlation between the two pollutants. The estimates of greenness and temperature were drawn from a model adjusted for NO_2_ (the best fit model), median age, concentration of migrant women (high vs low concentration), population density, and neighbourhood socioeconomic status. *denotes significant association.*

**Table S11: Summary of E-values for the association of different natural environment measures and GDM risk.**

| **Natural environment attributes** | **E-value for point estimate** | **E-value for the confidence interval** |
| --- | --- | --- |
| PM_2.5_ concentration | 1.00 | 1.00 |
| NO_2_ concentration | 1.11 | 1.00 |
| Residential greenness (Normalised Difference Vegetation Index [NDVI]) | 1.5 | 1.29 |
| Temperature (LST) | 1.11 | 1.00 |

The E-values for each exposure variable were calculated based on the point and CrIs estimates (Adjusted Risk Ratio [ARR]) using the spatiotemporal Model 3a-b outputs (Table S4) and Figure 1 in the main document.

**Table S12: Sensitivity analysis showing the association between natural environment and GDM risk at the SA3 level (checking modifiable areas unit problem [MAUP])**

| **Natural environment attributes** | **Adjusted Risk Ratio (ARR) (CrIs)** |
| --- | --- |
| PM_2.5_ concentration | 1.00(0.99, 1.02) |
| NO_2_ concentration | 1.02(0.99, 1.04) |
| Residential greenness (a 0.10 increase in NDVI) | **0.86 (0.79, 0.95)*** |
| Temperature (LST) | 0.98 (0.99, 1.00) |

**denotes significant association.*

**Table S13: Sensitivity analysis using the residual-exposure approach.**

| **Models** | **Exposures, pollutants** | **Exposures residualised** | **ARR (95% CrIs)** |
| --- | --- | --- | --- |
| 1 | NO_2_ | PM_2.5_ residuals | 0.99(99, 1.00) |
| 2 | PM_2.5_ | NO_2_ residuals | 1.02(0.99, 1.04) |

*The model is based on BYM-linear*

Table S14: Sensitivity analysis showing the association between the lag association of environmental exposures and GDM risk.

| **Natural environment attributes** | **Adjusted Risk Ratio (ARR) (CrIs)** |
| --- | --- |
| **Model: lag0(based on BYM2-linear)** |  |
| PM_2.5_ concentration | 1.01(1.00, 1.01) |
| NO_2_ concentration | 1.00(1.00, 1.01) |
| Residential greenness (a 0.10 increase in NDVI) | 1.05 (0.97, 1.13) |
| Temperature (LST) | 1.00 (0.99, 1.01) |
| **Model: lag1(based on BYM2-linear)** |  |
| PM_2.5_ concentration | 1.01(1.00, 1.02) |
| NO_2_ concentration | 1.01(0.99, 1.01) |
| Residential greenness (a 0.10 increase in NDVI) | 0.99 (0.91, 1.08) |
| Temperature (LST) | 1.00(0.99, 1.00) |

**Text S8: Codes for non-spatial (GLMM) and spatiotemporal models**

The analysis in this study was conducted using R version 4.3.0 programming language. A detailed methodological description is provided in Text S5 for GLMM and the ‘data analysis’ section of the manuscript for spatiotemporal ecological regression models. Variable labels with descriptions are provided in Table S8 below.

**Table S15: Variable labels and expressions.**

| **Variable label** | **Expression** |
| --- | --- |
| *Cases* | Number of GDM cases per SA2 in each year, 2016-2022 |
| *Year* | The year that ranged from 2016 to 2022 |
| *SES* | Socioeconomic status measured using the Socio-Economic Index for Area (SEIFA). The quintile was used based on the national standard classification approach for neighbourhood socioeconomic status. |
| *MedianAge* | Women’s median age in each SA2 |
| LQ_mig | Concentration of non-European migrant women per SA2 calculated using location quotient (LQ), as explained in the methods section. |
| Pop_den | Population density per SA2 |
| NDVI_med | Median normalized difference vegetation index per SA2 |
| Pm_2.5__med | Median PM_2.5_ concentration per SA2 |
| LST_med | Median Land surface temperature (a measure of ambient temperature) per SA2 |
| NO_2__med | Median NO_2_ concentration per SA2 |
| *SA2code* | Statistical Area Level 2 code |
| *offset(log(Pop))* | The logarithm of population (number of women who gave birth per SA2 per year) to account for population variation |
| *Ex* | Expected cases per SA2 |

**Text S9: R codes for the non-spatial and spatiotemporal models**

**Codes for negative binomial GLMM (Tables S5):**

***Model 1 (Area [SA2] only as a random effect****:*

nb.glmm1=glmmTMB(Cases~Year+SES+MedianAge+LQ_mig+Pop_den+NDVI_med+NO_2__med+LST_med+offset(log(Pop))+(1|SA2code),data = GDM_NE, family = nbinom1)

***Model 2 (Area [SA2] and time (Year) as random effects)****:*

nb.glmm1=glmmTMB(Cases~Year+SES+MedianAge+LQ_mig+Pop_den+NDVI_med+NO_med+LST_med+offset(log(Pop))+offset(log(Pop))+(1|SA2code)+(1|Year),data = GDM_NE, family = nbinom1)

**Codes for the negative binomial spatiotemporal ecological regression (Table S7, Model 3a-b**):

-IDarea and IDarea1: Codes for SA2 and we assigned them twice, as we specified twice in the model formulation (see below).

**-**BYM2: Besag-York-Mollie model

**-**IID: Independent and Identically Distributed model

**-**Ex: Expected count

**-**NbMt: The neighbourhood matrix (W)

-INLA: Integrated Laplace Approximation

-GDM_NE: The dataset

***Model 3a (single pollutant model, NO_2_)***

ST.NEFor1=Cases~SES+MedianAge+LQ_mig+Pop_den+NDVI_med+NO2_med+LST_med+f(IDarea, model = "bym2", graph = nbr, constr = TRUE)+f(IDarea1, Year, model = "iid")+Year1

Model 1=inla(ST.NEFor1, family = "nbinomial", data = GDM_NE, E=Ex, control.predictor = list(compute=TRUE), control.compute = list(waic=TRUE, dic=TRUE, cpo=TRUE, return.marginals.predictor = TRUE), verbose = TRUE)

***Model 3a (single pollutant model, PM_2.5_)***

ST_Form1=Cases~SES+MedianAge+LQ_mig+Pop_den+NDVI_med+pm2.5_med+LST_med+f(IDarea, model = "bym2", graph = NbMt, constr = TRUE)+f(IDarea1, Year, model = "iid")+Year1

Model 1=inla(ST_Form1, family = "nbinomial", data = GDM_NE, E=E, control.predictor = list(compute=TRUE), control.compute = list(waic=TRUE, dic=TRUE, cpo=TRUE, return.marginals.predictor = TRUE), verbose = TRUE)

**References**

1. Australian Bureau of Statstics. (2018). Census of Population and Housing: Socio-Economic Indexes for Areas (SEIFA), Australia, 2016. <https://www.abs.gov.au/statistics/people/people-and-communities/socio-economic-indexes-areas-seifa-australia/latest-release> [Accessed Date: 10 August].

2. Sydes M: Where immigrants live: capturing ethnic segregation at the local level in two Australian cities. *Australian Geographer* 2019, 50(2):221–241.doi: <https://doi.org/10.1080/00049182.2018.1512184>

3. Australian Bureau of Statistics. (2016). Australian Statistical Geography Standard (ASGS): Volume 1 - Main Structure and Greater Capital City Statistical Areas. <https://www.abs.gov.au/ausstats/abs@.nsf/Lookup/by%20Subject/1270.0.55.001~July%202016~Main%20Features~ABS%20structures~10007> [Accessed Date: 22 April 2024].

4. Ramsey S, Mavoa S: Annual Normalized Difference Vegetation Index time-series data for Australian statistical areas. 2021

5. Google Earth Engine. (2024). Landsat 8. <https://developers.google.com/earth-engine/datasets/catalog/landsat-8> [Accessed Date: 04 September].

6. Avdan U, Jovanovska G: Algorithm for automated mapping of land surface temperature using LANDSAT 8 satellite data. *Journal of sensors* 2016, 2016(1):1480307

7. Wang S, Cai W, Tao Y, Sun QC, Wong PPY, Huang X, Liu Y: Unpacking the inter- and intra-urban differences of the association between health and exposure to heat and air quality in Australia using global and local machine learning models. *Sci Total Environ* 2023, 871:162005.doi: 10.1016/j.scitotenv.2023.162005

8. Dunn PK, Smyth GK, Dunn PK, Smyth GK: Chapter 10: Models for Counts: Poisson and Negative Binomial GLMs. *Generalized Linear Models With Examples in R* 2018:371–424

9. Moran PA: Notes on continuous stochastic phenomena. *Biometrika* 1950, 37(1/2):17–23

10. Moraga Paula: Spatial statistics for data science: theory and practice with R; 2023.

11. Duncan EW, White NM, Mengersen K: Spatial smoothing in Bayesian models: a comparison of weights matrix specifications and their impact on inference. *International journal of health geographics* 2017, 16:1–16

12. Moraga Paula: Spatial Statistics for Data Science: Theory and Practice with R. Chapman & Hall/CRC Data Science Series.; R. CRC Press, 2023. <https://www.paulamoraga.com/book-geospatial/index.html>

13. Blangiardo M, Cameletti M, Baio G, Rue H: Spatial and spatio-temporal models with R-INLA. *Spat Spatiotemporal Epidemiol* 2013, 7:39–55.doi: 10.1016/j.sste.2013.07.003

14. Blangiardo M, Cameletti M: Spatial and spatio-temporal Bayesian models with R-INLA; 2015.

15. Gómez-Rubio V: Bayesian Inference with INLA. ; 2020. <https://becarioprecario.bitbucket.io/inla-gitbook/index.html>
